# Induction of ferroptosis in response to graphene quantum dots through mitochondrial oxidative stress in microglia

**DOI:** 10.1186/s12989-020-00363-1

**Published:** 2020-07-11

**Authors:** Tianshu Wu, Xue Liang, Xi Liu, Yimeng Li, Yutong Wang, Lu Kong, Meng Tang

**Affiliations:** 1 Key Laboratory of Environmental Medicine and Engineering, Ministry of Education; School of Public Health, Southeast University, Nanjing, 210009 P. R. China; 2 School of Medicine, Southeast University, Nanjing, 210009 P. R. China

**Keywords:** ROS, Iron overload, GSH depletion, Lipid peroxidation, Mitochondrial dysfunction

## Abstract

**Background:**

Graphene quantum dots (GQDs) provide a bright prospect in the biomedical application because they contain low-toxic compounds and promise imaging of deep tissues and tiny vascular structures. However, the biosafety of this novel QDs has not been thoroughly evaluated, especially in the central nervous system (CNS). The microarray analysis provides a hint that nitrogen-doped GQDs (N-GQDs) exposure could cause ferroptosis in microglia, which is a novel form of cell death dependent on iron overload and lipid peroxidation.

**Results:**

The cytosolic iron overload, glutathione (GSH) depletion, excessive reactive oxygen species (ROS) production and lipid peroxidation (LPO) were observed in microglial BV2 cells treated with N-GQDs, which indicated that N-GQDs could damage the iron metabolism and redox balance in microglia. The pre-treatments of a specific ferroptosis inhibitor Ferrostatin-1 (Fer-1) and an iron chelater Deferoxamine mesylate (DFO) not only inhibited cell death, but also alleviated iron overload, LPO and alternations in ferroptosis biomarkers in microglia, which were caused by N-GQDs. When assessing the potential mechanisms of N-GQDs causing ferroptosis in microglia, we found that the iron content, ROS generation and LPO level in mitochondria of BV2 cells all enhanced after N-GQDs exposure. When the antioxidant ability of mitochondria was increased by the pre-treatment of a mitochondria targeted ROS scavenger MitoTEMPO, the ferroptotic biological changes were effectively reversed in BV2 cells treated with N-GQDs, which indicated that the N-GQDs-induced ferroptosis in microglia could be attributed to the mitochondrial oxidative stress. Additionally, amino functionalized GQDs (A-GQDs) elicited milder redox imbalance in mitochondria and resulted in less ferroptotic effects than N-GQDs in microglia, which suggested a slight protection of amino group functionalization in GQDs causing ferroptosis.

**Conclusion:**

N-GQDs exposure caused ferroptosis in microglia via inducing mitochondrial oxidative stress, and the ferroptotic effects induced by A-GQDs were milder than N-GQDs when the exposure method is same. This study will not only provide new insights in the GQDs-induced cell damage performed in multiple types of cell death, but also in the influence of chemical modification on the toxicity of GQDs.

## Background

With the rapid development and application of graphene quantum dots (GQDs) that are considered as a novel type of QDs consisting of carbon atoms, their exposure to the public and environment is increasing in recent years. Since the size of GQDs is less than 20 nm, it is easy for them to reach and accumulate in the central nervous system (CNS) through bypassing the blood-brain barrier and result in severe neuronal disorders [1, 2]. Otherwise, the unique photoluminescence (PL) properties of GQDs with good biocompatibility enable them be an excellent fluorescent probes for bioimaging and biosensing of neurodegenerative disorders [3]. Therefore, it is an emerging research topic regarding the risks of GQDs in the CNS. Recently, researchers have demonstrated the contribution of GQDs to the diagnosis, prevention and treatment of brain diseases [2, 4, 5]. However, since most studies about GQDs in the CNS are involved in the application rather than the toxicity, the rare data from the neurotoxicological studies on GQDs will reversely impose restrictions on the application.

Recently, nitrogen-doped GQDs (N-GQDs) becomes a promising GQDs in environmental and biological applications because doping of nitrogen enhances the photochemical activity of GQDs for clear images with a high signal to noise ratio [3, 6, 7]. However, the toxicity studies on N-GQDs are underway and have not yet reached a consistent conclusion. Even though some researchers have suggested N-GQDs have low cytotoxicity [8], others show N-GQDs could be internalized into cells and disrupt some enzyme activities [9]. The N-GQDs, as fluorescent nanoprobes, could contribute to the development of nanotheranostics in the field of neuronscience due to their excellent optical properties, so it is significant to evaluate the interaction between N-GQDs and neurobiological models before applying them in living organisms.

The rich in unsaturated fatty acids for the generation of lipid reactive oxygen species (ROS) makes brain susceptible to redox imbalance [10]. The products of lipid peroxidation (LPO) caused by nanoparticles could resulted in severe brain damage, such as neurodegenerative disorders, and the ferroptosis with involvement of iron metabolism and LPO is defined as a critical form of cell death in neurodegenerative disorders [11, 12]. In reality, there are mixed forms of cell death rather than a certain single mode involved in the cell death induced by nanoparticles [13]. Ferroptosis, as a novel programmed cell death (PCD) named in 2002 [14], has been found in cultured cells and living animals exposed to some nanoparticles, including carbon ones [15–17]. Although nanoparticle-induced cell death through the ferroptosis pathway provides insights to advance nano-based and tumor-targeted theranostics, it increases risk on the normal tissues.

Microglia are critical CNS-specific cells playing a critical role in the development and homeostasis of brain, and glial cell death participates in many brain diseases [18]. Evaluating the harmful effects of nanoparticles in microglia is an important part of risk assessment of nanoparticles in the CNS. The microarray data suggested the expressions of most genes associated with ferroptosis, especially some biomarker genes, significantly altered in microglia treated with N-GQDs. Along with the consistent findings of several types of QDs exposure inducing oxidative stress that is considered as a typical characteristic of ferroptosis, it is reasonable to hypothesize ferroptosis is a novel mechanism by which GQDs induce toxicity in microglia. In this study, apart from observing accumulation of iron, GSH depletion and redox imbalance in microglia exposed to N-GQDs, two reported ferroptosis inhibitors, i.e. a lipid peroxidation inhibitor Ferrostatin-1 (Fer-1) and an iron chelator Deferoxamine mesylate (DFO), preventing microglia from cell death caused by N-GQDs confirmed the induction of ferroptosis in response to N-GQDs. We also used total antioxidant Trolox and mitochondria targeted antioxidant Mito-TEMPO to find that ferroptosis in microglia is mainly attributed to the mitochondrial oxidative stress caused by N-GQDs.

## Results

### The characterisation of N-GQDs and A-GQDs

**Fig. 1:**
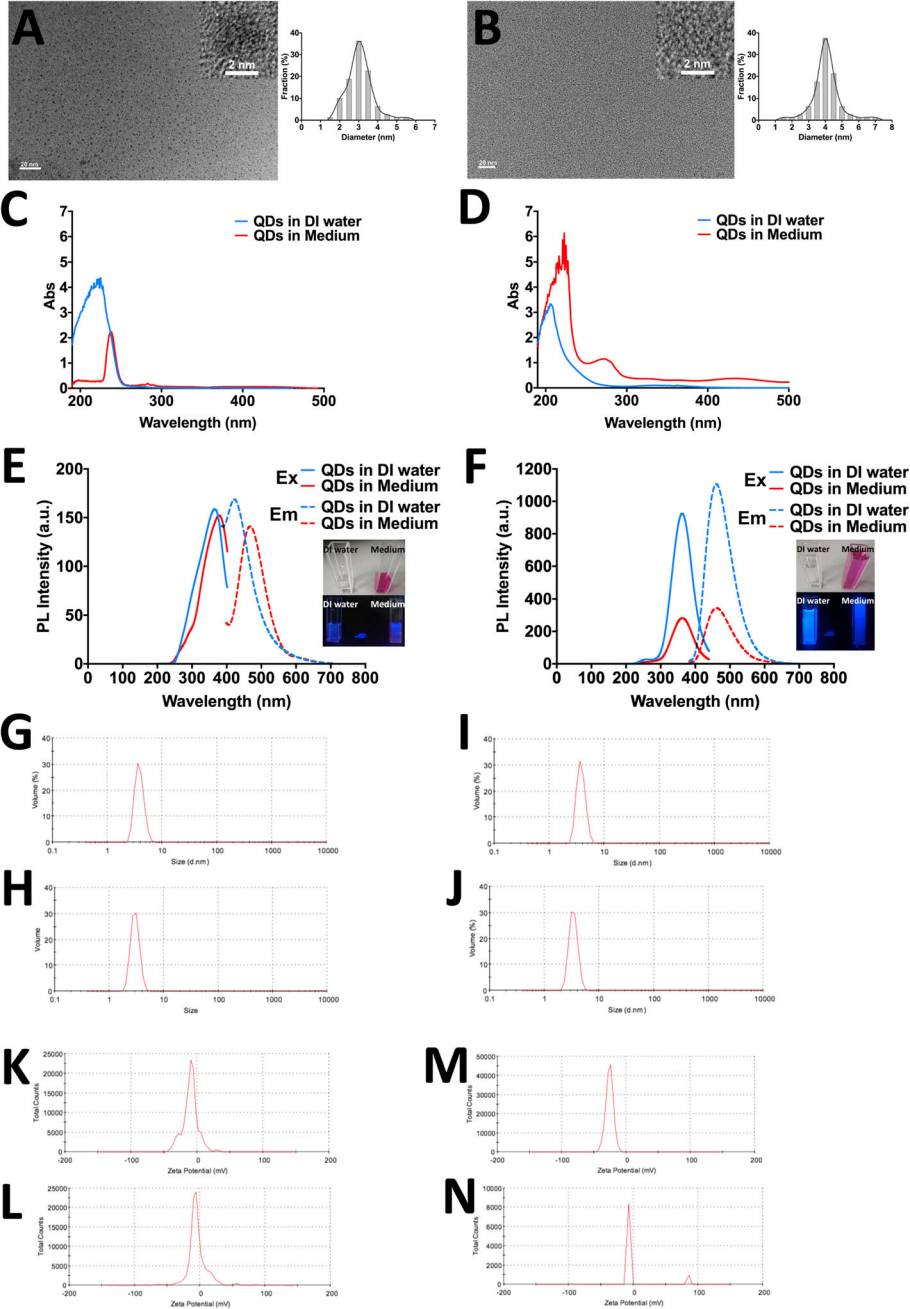
The Physicochemical characterization of N-GQDs and A-GQDs. **a**, **b** TEM imaging and size distribution of N-GQDs and A-GQDs, respectively; **c**, **d** UV-vis absorption spectra of N-GQDs and A-GQDs in deionized (DI) water and culture medium; **e**, **f** Fluorescence spectra of N-GQDs and A-GQDs in DI water and culture medium, emission spectrum of N-GQDs at λ_ex_ = 350 nm for DI water and λ_ex_ = 380 nm for medium, and excitation spectrum of N-GQDs at λ_em_ = 420 nm for DI water and λ_em_ = 480 nm for medium, while emission spectrum of A-GQDs at λ_ex_ = 380 nm for both DI water and medium, and excitation spectrum of A-GQDs at λ_em_ = 460 nm for both DI water and medium, respectively. Insert: Photographs of N-GQDs and A-GQDs in DI water or culture medium under the radiation of white light (Up) and 365 nm UV lamp (Down); Hydrous particle diameter of N-GQDs (**g**, **h**) and A-GQDs (**i**, **j**) in DI water and culture medium, respectively; Zeta potential of N-GQDs (**k**, **l**) and A-GQDs (**m**, **n**) in DI water and culture medium, respectively

The representative TEM images suggested both individual N-GQDs and A-GQDs were uniform with an average particle size of approximately 3 nm and 4 nm, respectively (Fig. 1a and b), and the lattice structure of N-GQDs seemed better than A-GQDs. In order to accord with the cell culture environment, the optical and physical properties of two types of GQDs were assessed when they are dissolved in culture medium and deionized (DI) water at the concentration of 100 μg/mL. The absorbance spectra indicated that the UV-vis absorption peaks of N-GQDs and A-GQDs both had a red-shifted emission when the solution diluting GQDs was from DI water shifting into cell medium, but they still emitted in the blue spectral region (Fig. 1c and d).

Both two types of GQDs exhibited obvious and narrow fluorescence spectra when assessing their photoluminescence (PL). The emission peak and excitation peak of N-GQDs in culture medium centered at the wavelength slightly larger than that in DI water, but the fluorescent intensities were similar in water and in medium (Fig. 1e). On the contrary, the emission peak and excitation peak of A-GQDs centered at the same place in culture medium and DI water, while the fluorescent intensity in water was obviously stronger than that in medium (Fig. 1f). Meanwhile, the diluted solutions of N-GQDs and A-GQDs in DI water and cell medium were almost colorless observed by the naked eyes under ambient daylight, and presented bright blue fluorescence under the 465 nm UV light, but the fluorescence of A-GQDs in DI water was obviously stronger than that in medium (Fig. 1e and f). According to the quality reports from manufacture, the photoluminescence quantum yields (PLQYs) of N-GQDs and A-GQDs were both approximately 20% (Table S2).

With regard to the hydrodynamic diameter of GQDs, the mean sizes of N-GQDs in DI water and in culture medium were similar (Fig. 1g and h), but both slightly larger than that measured by TEM, while the mean sizes of A-GQDs in DI water and in culture medium were similar as well (Fig. 1i and j), but both were slightly smaller than that measured by TEM (Table S2). Both two types of GQDs were negatively charged in DI water and in culture medium, but the zeta potentials of N-GQDs in culture medium were more negative than in DI water (Fig. 1k and l), while the zeta potentials of A-GQDs in culture medium were less negative than in DI water (Fig. 1m and n), which was probably attributed to the amino-group on the particle surface interacting with some components in culture medium.

### The internalization of N-GQDs induced ferroptosis in microglia

**Fig. 2:**
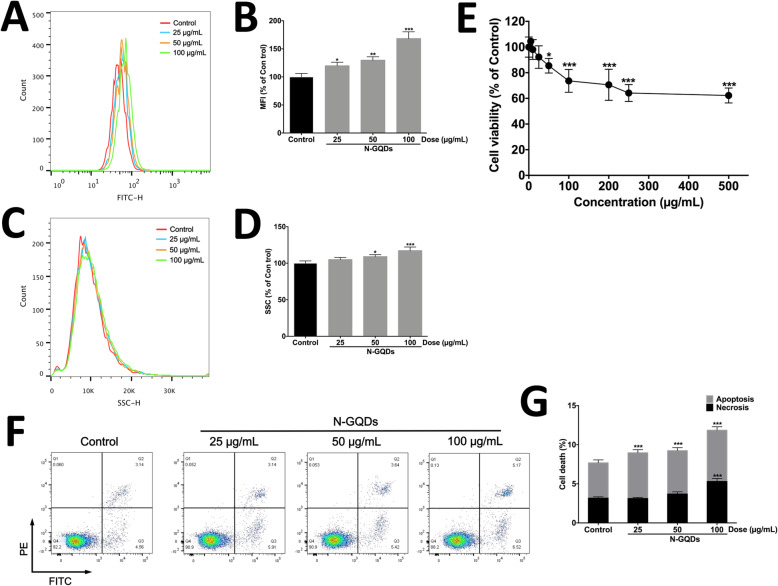
The cytotoxicity of N-GQDs in BV2 cells. Representative FITC fluorescence histogram plot (**a**) and side scattering (SSC) histogram plot (**c**) of BV2 cells in presence of 25, 50 and 100 μg/mL N-GQDs for 24 h under a flow cytometer at excitation and emission wavelengths of 488 nm and 525 nm; Quantitative results of mean fluorescence intensity (MFI) (**b**) and SSC relative to control (**d**) from flow cytometer analysis; **e** The effects of N-GQDs on cell viability in BV2 cells at the concentrations ranging from 5 ~ 500 μg/mL for 24 h were measured by CCK8 assay; **f** Representative flow cytometer dot plot of apoptosis and necrosis in BV2 cells treated with 25, 50 and 100 μg/mL N-GQDs for 24 h were identified by using Annexin V-FITC/PI; **g** Quantitative results of necrotic and apoptotic percentages from flow cytometer analysis. Data are expressed as the mean ± SE of three independent experiments, performed in triplicate. Statistical significance was determined by one-way ANOVA and Dunnett’s t test (*P < 0.05, **P < 0.01, ***P < 0.001 vs. the control group)

Since cell parameters can change in the presence of N-GQDs due to their size and bright fluorescent, the flow cytometry provides a fast and reliable method to analyze nanoparticle internalization into cells [19]. In this study, the mean fluorescent intensity (MFI) quantification and side-scattered light (SSC) related to cell internal complexity in BV2 cells showed a dose-dependent increase after N-GQDs treatment for 24 h (Fig. 2a~d). The data suggested that N-GQDs exposure induced a dose-dependent internalization in BV2 cells evidenced by the enhancement in fluorescence (MFI) and intracellular granularity (SSC).

The cell viability of BV2 cells treated with N-GQDs is decreasing along with the increasing administration concentration (Fig. 2e). The decreased cell viability became significantly different from the control when the concentration is above 50 μg/mL. After that, the cell viability fall to approximately 60% and became stable when the concentration is over 250 μg/mL. According to some reported studies showing administrated doses for biomedical applications in cultured cells and model animals [20–22] as well as the results of CCK8 assay in this study, the treatment concentrations of N-GQDs in following experiments are 25, 50 and 100 μg/mL. When distinguishing the apoptosis and necrosis caused by N-GQDs in BV2 cells, the data showed that N-GQDs treatment with concentration above 25 μg/mL caused a significantly increase in the quantity of apoptotic cells, while only 100 μg/mL N-GQDs treatment significantly increased the quantity of necrotic cells when compared to the control (Fig. 2f and g).

**Fig. 3:**
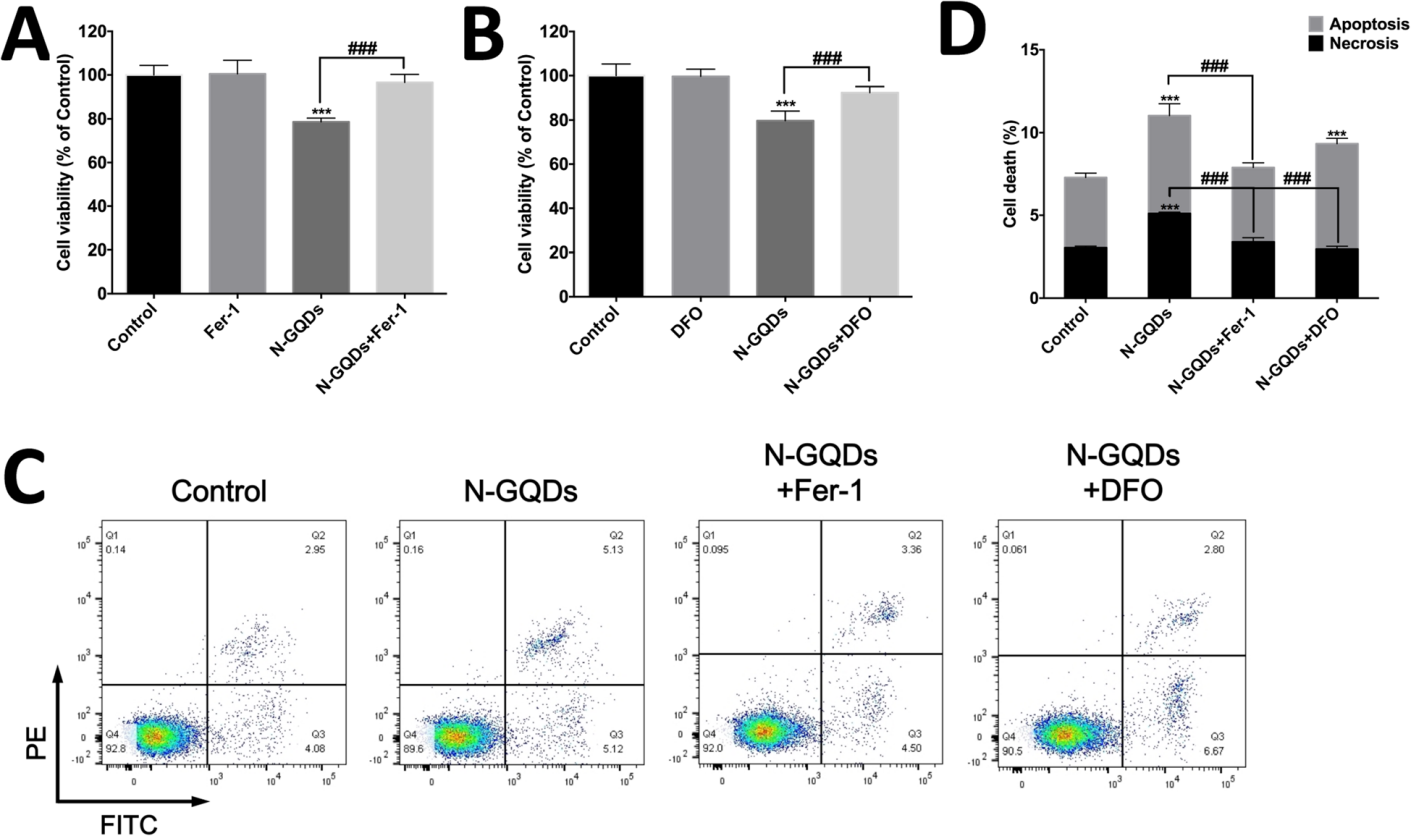
N-GQDs caused ferroptosis in BV2 cells. The cell viability of BV2 cells treated with 100 μg/mL N-GQDs for 24 h pretreated with/without a specific ferroptosis inhibitor Fer-1 (**a**) and an iron chelator DFO (**b**) were measured by CCK8 assay; **c** Representative flow cytometer dot plot of apoptosis and necrosis in BV2 cells treated with 100 μg/mL N-GQDs for 24 h pretreated with/without Fer-1 and DFO were identified by using Annexin V-FITC/PI; **d** Quantitative results of necrotic and apoptotic percentages from flow cytometer analysis. Data are expressed as the mean ± SE of three independent experiments, performed in triplicate. Statistical significance was determined by one-way ANOVA and Dunnett’s t test (*P < 0.05, **P < 0.01, ***P < 0.001 vs. the control group; #*P* < 0.05, ##*P* < 0.01, ###*P* < 0.001 vs. the 100 μg/mL N-GQDs group)

In order to assess the involvement of ferroptosis in cell death caused by N-GQDs, two specific ferroptosis inhibitors, i.e. Fer-1 and DFO, was used to pre-treat BV2 cells. Fer-1 is a lipid peroxidation inhibitor and DFO is an iron chelator, which are both reported to block ferroptosis in different types of cells induced by various ferroptosis inducers [14, 23, 24]. The data showed that decreased cell viability of BV2 cells treated with 100 μg/mL N-GQDs was inhibited by both Fer-1 and DFO (Fig. 3a and b). Additionally, Fer-1 and DFO also completely protected BV2 cells from necrosis caused by N-GQDs, while Fer-1 also protected BV2 cells from N-GQDs-induced apoptosis (Fig. 3c and d).

### N-GQDs exposure elicited characteristic changes of ferroptosis in microglia, including cytosolic iron overload, GSH depletion and lipid peroxidation

**Fig. 4:**
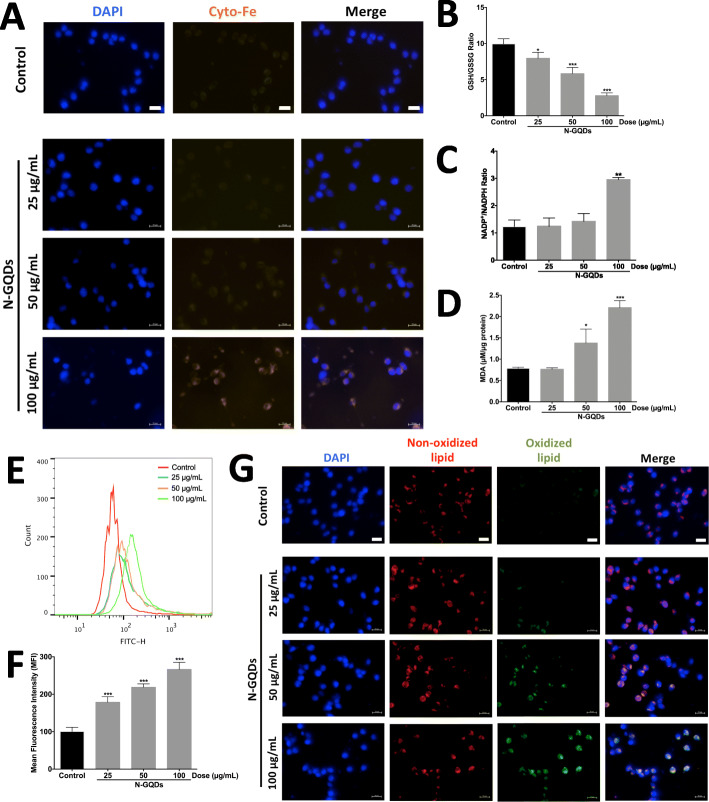
N-GQDs damaged the iron metabolism and redox balance in BV2 cells. **a** Representative fluorescent images of intracellular iron level in BV2 cells treated with 25, 50 and 100 μg/mL N-GQDs for 24 h were identified by using FerroOrange (orange). Nuclei are stained by DAPI (blue). Scale bars: 50 μm; The effects of 25, 50 and 100 μg/mL N-GQDs treatments for 24 h on GSH/GSSG ratio (**b**), NADP^+^/NADPH ratio (**c**) and MDA content (**d**) in BV2 cells were measured by ELISA; **e** Representative FITC fluorescence histogram plot of cytosolic ROS production in BV2 cells treated with 25, 50 and 100 μg/mL N-GQDs for 24 h were identified by using DCFH-DA; **f** Quantitative results of mean fluorescence intensity (MFI) from flow cytometer analysis; **g** Representative fluorescent images of lipid ROS in BV2 cells treated with 25, 50 and 100 μg/mL N-GQDs for 24 h were identified by using C11BODIPY^581/591^. Nonoxidized lipid is in red, oxidized lipid is in green, nuclei are stained by DAPI (blue), merging of the red and green color results in a yellow signal. Scale bars: 50 μm. Data are expressed as the mean ± SE of three independent experiments, performed in triplicate. Statistical significance was determined by one-way ANOVA and Dunnett’s t test (*P < 0.05, **P < 0.01, ***P < 0.001 vs. the control group)

Firstly, we confirmed the blue fluorescence of N-GQDs could be ignored when cells are stained by DAPI to mark nucleus (Fig. S1). As the iron overload is a major character of ferroptosis, the levels of cytosolic iron contents were measured in BV2 cells treated with N-GQDs, and representative images showed iron levels in cytoplasm identified by FerroOrange probes obviously increased following with the increasing administration concentrations (Fig. 4a). Meanwhile, pre-treatments of Fer-1 and DFO both remarkably lessen the excessive cytosolic iron contents in BV2 cells that were ignited by 100 μg/mL N-GQDs (Fig. S2).

Since the redox imbalance in cells has been reported to contribute to the occurrence of ferroptosis [14, 24], some indexes indicating redox homeostasis disruption were detected in BV2 cells treated with N-GQDs. As tripeptide GSH serving a protective role on antioxidant defence, the data showed the ratio of GSH and GSSG significantly deceased after N-GQDs treatment for 24 h when compared to the control, which indicated that N-GQDs induced GSH depletion in BV2 cells in a dose-dependent manner (Fig. 4b). The ratio of NADP^+^ and NADPH significantly increased in cells treated with N-GQDs at the high dose (Fig. 4c), and this decreased NADPH level was associated with GSH depletion because the NADPH was a coenzyme of GSH reductase that participates in maintaining the GSH content. Meanwhile, the end-product of LPO, i.e. MDA contents and ROS production both enhanced in BV2 cells treated with N-GQDs for 24 h (Fig. 4D~f). Apart from the MDA, the levels of oxidized lipid highlighted by the C11-BODIPY^581/591^ probe also increased in BV2 cells treated with concentrations above 50 μg/mL (Fig. 4g). Fortunately, N-GQDs-induced reduction in GSH level and increases in MDA contents, ROS generation and oxidative lipid were all completely blocked by pre-treatments of Fer-1 or DFO (Fig. S3).

### N-GQDs exposure alerted expression patterns of genes and proteins relevant to ferroptosis in microglia

**Fig. 5:**
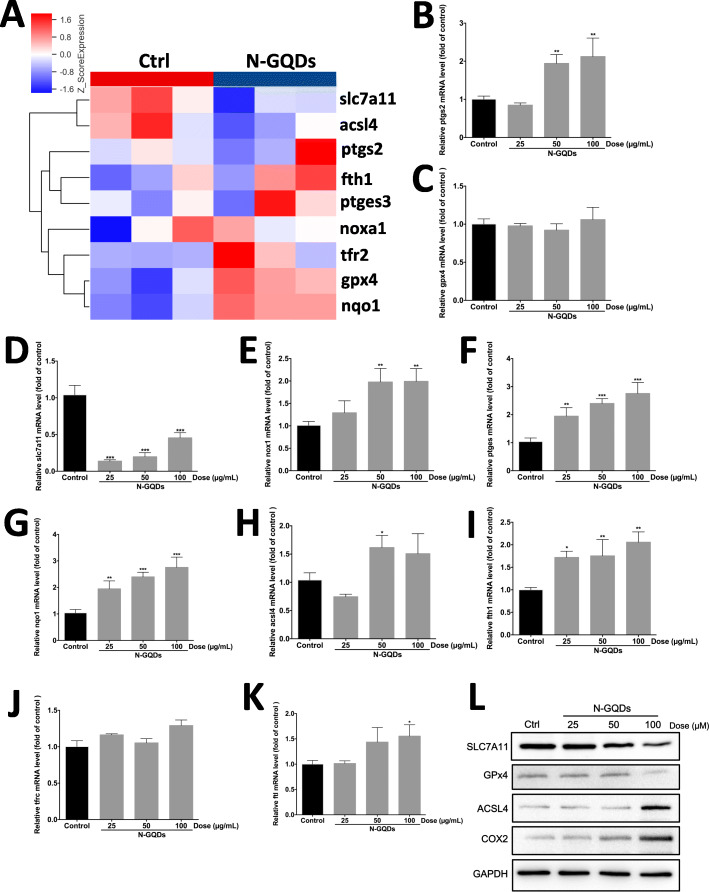
N-GQDs altered the expressions of ferroptosis-related genes and proteins in BV2 cells. **a** Differentially expressed genes associated with ferroptosis in BV2 cells treated with 100 μg/mL N-GQDs for 24 h were detected by microarray; The expressions of redox balance-related genes ptgs2 (**b**), gpx4 (**c**), slc7a11 (**d**), nox1 (**e**), ptges (**f**), nqo1 (**g**), acsl4 (**h**) and iron metabolism-related genes fth1 (**i**), tfrc (**j**), ftl (**k**) in BV2 cells treated with 25, 50 and 100 μg/mL N-GQDs for 24 h were determined by qRT-PCR analysis; **l** The expressions of ferroptosis biomarker proteins SLC7A11, GPX4, ACSL4 and COX2 in BV2 cells treated with 25, 50 and 100 μg/mL N-GQDs for 24 h were determined by western blotting analysis. Data are expressed as the mean ± SE of three independent experiments, performed in triplicate. Statistical significance was determined by one-way ANOVA and Dunnett’s t test (*P < 0.05, **P < 0.01, ***P < 0.001 vs. the control group)

The results of mRNA microarray assay indicated some differentially expressed genes between 100 μg/mL N-GQDs-treated cells and the control were enriched in the pathway associated with ferroptosis (Fig. 5a). The results of qRT-PCR analysis also confirmed that the expression levels of genes ptgs2, slc7a11, nox1, ptges, nqo1, acsl4, fth1 and ftl altered in the BV2 cells treated with 25 to 100 μg/mL N-GQDs to some extent (Fig. 5b~k). Additionally, the changed protein expression of four widely-reported biomarkers of ferroptosis, i.e. SLC7A11, GPX4, ACSL4 and COX2 in cells were measured after N-GQDs treatment. The results suggested that 100 μg/mL N-GQDs obviously inhibited the expression levels of protein SLC7A11 and GPx4, while enhanced that of protein ACSL4 and COX2 (Fig. 5l), and the alternation in expression patterns of four proteins can be reversed by pre-treatment of Fer-1 and DFO (Fig. S4).

### Mitochondrial damage induced by N-GQDs contributed to the ferroptosis in microglia

**Fig. 6:**
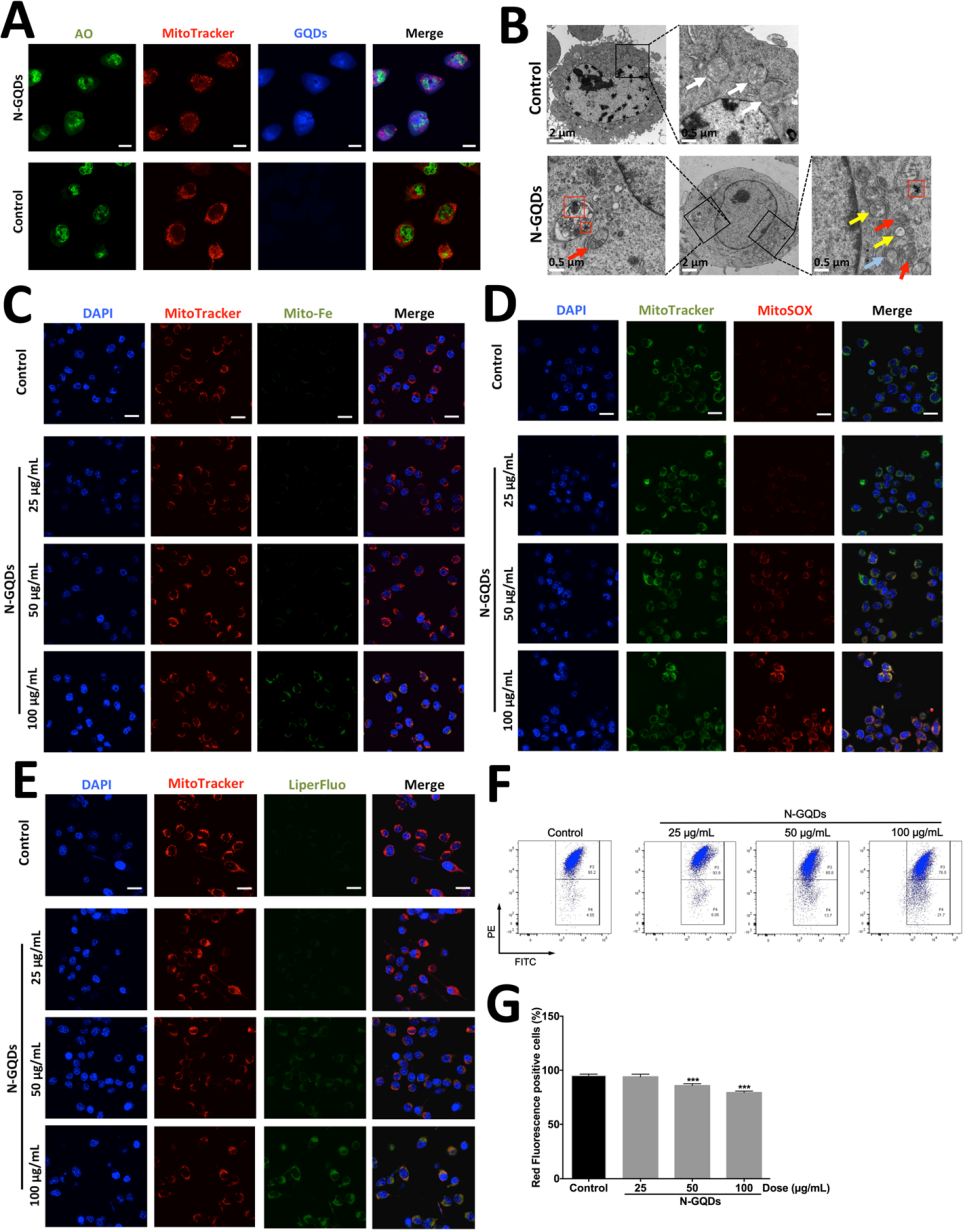
N-GQDs caused oxidative stress and dysfunction in mitochondria of BV2 cells. **a** Representative fluorescent images of internalization of N-GQDs (blue) in BV2 cells treated with 100 μg/mL N-GQDs for 24 h. Nuclei are stained by AO (green). Mitochondria are stained by MitoTracker (red). Scale bars: 10 μm; **b** TEM images showing the ultrastructure of BV2 cells treated with 100 μg/mL N-GQDs for 24 h. The asterisks indicate aggregated GQDs in endosomes/lysosomes. The red boxes indicate aggregated GQDs. The white arrows indicate normal mitochondria, the red arrows indicate mitochondrial shrinkage, the yellow arrows indicate broken mitochondrial ridge, and the blue arrows indicate collapse of mitochondrial membrane; Representative fluorescent images of mitochondrial iron level (**c**), mitochondrial ROS (mtROS) production (**d**) and mitochondrial lipid peroxidation (**e**) in BV2 cells treated with 25, 50 and 100 μg/mL N-GQDs for 24 h. Nuclei are stained by DAPI (blue). Iron levels are detected by Mito-FerroGreen (green). mtROS are detected by MitoSOX (red). Oxidative lipid are detected by LiperFluo (green). Mitochondria are marked by MitoTracker (red) and MitoTracker (green), respectively. Merging of the red and green color results in a yellow signal. Scale bars: 20 μm; **f** Representative flow cytometer dot plot of mitochondrial transmembrane potential (Δψ_mt_) in BV2 cells treated with 25, 50 and 100 μg/mL N-GQDs for 24 h were identified by using JC-1; **g** Quantitative results of red fluorescence positive cells from flow cytometer analysis. Data are expressed as the mean ± SE of three independent experiments, performed in triplicate. Statistical significance was determined by one-way ANOVA and Dunnett’s t test (*P < 0.05, **P < 0.01, ***P < 0.001 vs. the control group)

Recently, researchers have found the important role of mitochondria in the induction of ferroptosis [25]. Firstly, the internalization and distribution of N-GQDs into BV2 cells at the concentration 100 μg/mL were confirmed by confocal microscopy. Representative images showed that internalized N-GQDs could accumulate in whole cells, including mitochondria (Fig. 6a), which might damage mitochondria directly. When observing the ultrastructure of BV2 cells treated with N-GQDs at the high dose through TEM, detailed impairments of mitochondria, including shrinkage, broken ridge and collapsed membrane, as well as aggregated GQDs were found (Fig. 6b). The iron levels, ROS production and oxidative lipid in mitochondria of BV2 cells were assessed in N-GQDs-treated and non-treated cells by using corresponding fluorescent probes, and these indexes associated with iron metabolism and LPO all increased after N-GQDs treatments for 24 h, especially 100 μg/mL GQDs (Fig. 6c~e). Meanwhile, the MMP in BV2 cells treated with 50 and 100 μg/mL N-GQDs significantly decreased when compared to the control (Fig. 6f and g), which indicated mitochondrial dysfunction caused by N-GQDs.

**Fig. 7:**
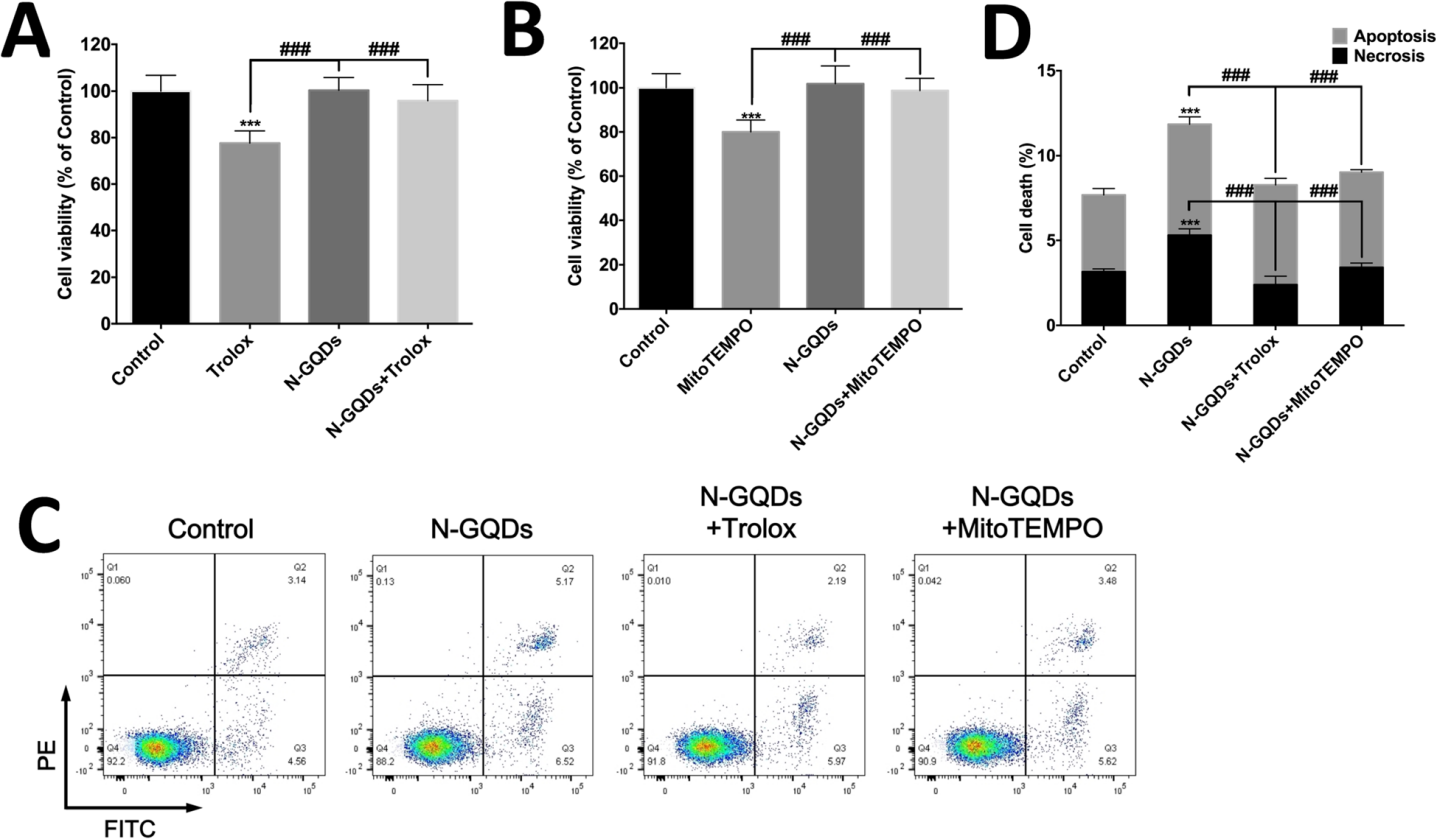
The ROS scavengers inhibited BV2 cell death caused by N-GQDs. The effect of a total antioxidant Trolox (**a**) and a mitochondria targeted ROS scavenger MitoTEMPO (**b**) on the cell viability in BV2 cells treated with 100 μg/mL N-GQDs for 24 h were measured by CCK8 assay; **c** Representative flow cytometer dot plot of apoptosis and necrosis in BV2 cells treated with 100 μg/mL N-GQDs for 24 h pretreated with/without Trolox and MitoTEMPO were identified by using Annexin V-FITC/PI; **d** Quantitative results of necrotic and apoptotic percentages from flow cytometer analysis. Data are expressed as the mean ± SE of three independent experiments, performed in triplicate. Statistical significance was determined by one-way ANOVA and Dunnett’s t test (*P < 0.05, **P < 0.01, ***P < 0.001 vs. the control group; #*P* < 0.05, ##*P* < 0.01, ###*P* < 0.001 vs. the 100 μg/mL N-GQDs group)

Since many studies have reported that excessive ROS production is a direct inducer of ferroptosis and mitochondria is the primary source of ROS [24, 26], we assess the protection of a total antioxidant Trolox and a mtROS scavenger MitoTEMPO on the BV2 cell death caused by 100 μg/mL N-GQDs treatment for 24 h after confirming their ROS scavenging capacity (Fig. S5). The data showed that Trolox and MitoTEMPO both completely protect BV2 cells from cell viability damaged by N-GQDs (Fig. 7a and b). Furthermore, Trolox and MitoTEMPO were capable of inhibiting both apoptosis and necrosis caused by N-GQDs (Fig. 7c and d).

**Fig. 8:**
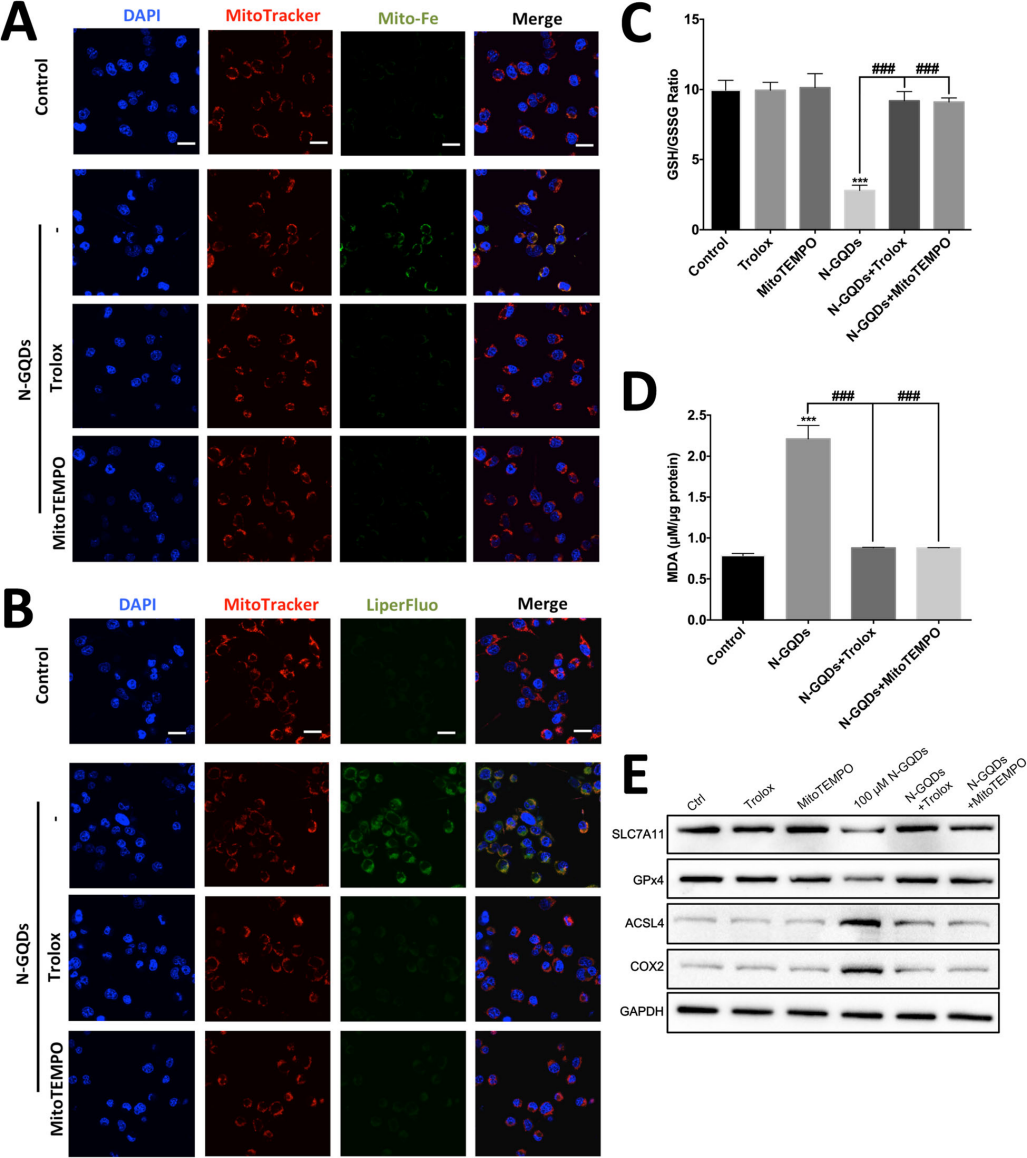
The ROS scavengers alleviated impairments of iron metabolism and redox balance caused by N-GQDs in BV2 cells. Representative fluorescent images of mitochondrial iron level (**a**) and mitochondrial lipid peroxidation (**b**) in BV2 cells treated with 100 μg/mL N-GQDs for 24 h pretreated with/without Trolox and MitoTEMPO. Nuclei are stained by DAPI (blue). Iron levels are detected by Mito-FerroGreen (green). Oxidative lipid are detected by LiperFluo (green). Mitochondria are marked by MitoTracker (red). Merging of the red and green color results in a yellow signal. Scale bars: 20 μm; The GSH/GSSG ratio (**c**) and the MDA content (**d**) in BV2 cells treated with 100 μg/mL N-GQDs for 24 h pretreated with/without Trolox and MitoTEMPO were measured by ELISA; **e** The expressions of ferroptosis marker proteins SLC7A11, GPX4, ACSL4 and COX2 in BV2 cells treated with 100 μg/mL N-GQDs for 24 h pretreated with/without Trolox and MitoTEMPO were determined by western blotting analysis. Data are expressed as the mean ± SE of three independent experiments, performed in triplicate. Statistical significance was determined by one-way ANOVA and Dunnett’s t test (*P < 0.05, **P < 0.01, ***P < 0.001 vs. the control group; #*P* < 0.05, ##*P* < 0.01, ###*P* < 0.001 vs. the 100 μg/mL N-GQDs group)

In addition, the iron overload and oxidative lipid in mitochondria caused by N-GQDs were inhibited effectively by pre-treatments of Trolox and MitoTEMPO (Fig. 8a and b), while two ferroptosis inhibitors can obviously inhibited mitochondrial iron overload and oxidative stress as well (Figs. S6 and S7). Trolox and MitoTEMPO also reversed the GSH depletion, MDA enhancement, and altered expression pattern of marker protein of ferroptosis caused by N-GQDs in BV2 cells (Fig. 8c~e). The findings indicated that cytosolic and mitochondrial ROS production scavenged by Trolox and MitoTEMPO could contribute to the ferroptosis induced by N-GQDs.

### A-GQDs exposure caused milder ferroptotic effects than N-GQDs in microglia attributed to slighter mitochondrial oxidative stress

**Fig. 9:**
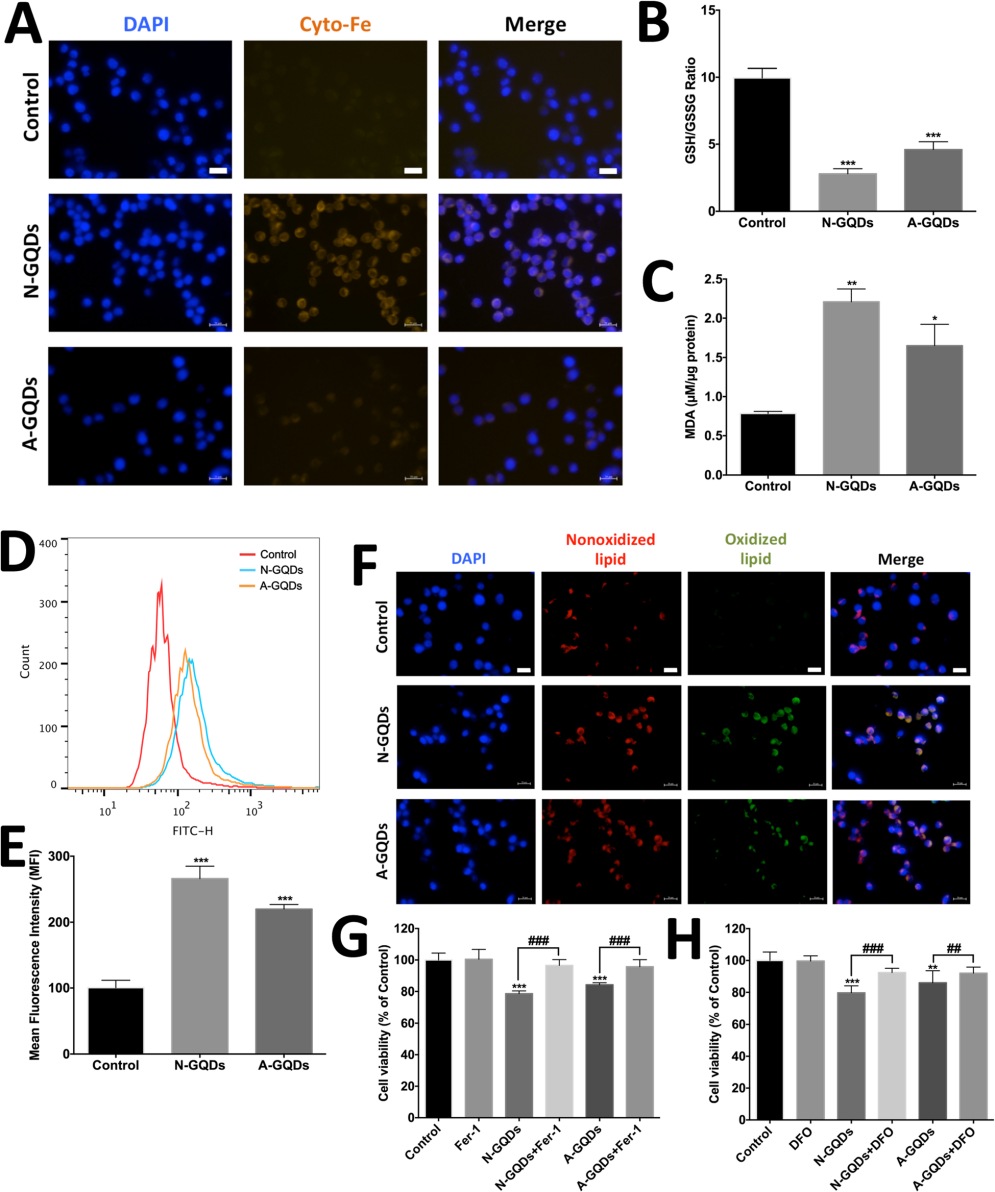
A-GQDs caused slighter ferroptosis-related damages in BV2 cells than N-GQDs. **a** Representative fluorescent images of intracellular iron level in BV2 cells treated with 100 μg/mL N-GQDs and 100 μg/mL A-GQDs for 24 h were identified by using FerroOrange (orange). Nuclei are stained by DAPI (blue). Scale bars: 50 μm; The effects of 100 μg/mL N-GQDs and 100 μg/mL A-GQDs treatments for 24 h on the GSH/GSSG ratio (**b**) and the MDA content (**c**) in BV2 cells were measured by ELISA; **d** Representative FITC fluorescence histogram plot of cytosolic ROS production in BV2 cells treated with 100 μg/mL N-GQDs and 100 μg/mL A-GQDs for 24 h were identified by DCFH-DA; **e** Quantitative results of mean fluorescence intensity (MFI) from flow cytometer analysis; **f** Representative fluorescent images of lipid peroxidation in BV2 cells treated with 100 μg/mL N-GQDs and 100 μg/mL A-GQDs for 24 h were identified by using C11BODIPY^581/591^. Nonoxidized lipid is represented in red, oxidized lipid is in green, nuclei are stained by DAPI (blue), merging of the red and green color results in a yellow signal. Scale bars: 50 μm; The effect of Fer-1 (**g**) and DFO (**h**) on the cell viability in BV2 cells treated with 100 μg/mL N-GQDs and 100 μg/mL A-GQDs for 24 h were measured by CCK8 assay. Data are expressed as the mean ± SE of three independent experiments, performed in triplicate. Statistical significance was determined by one-way ANOVA and Dunnett’s t test (*P < 0.05, **P < 0.01, ***P < 0.001 vs. the control group; #*P* < 0.05, ##*P* < 0.01, ###*P* < 0.001 vs. the 100 μg/mL N-GQDs group)

Amino group is a common chemical modification to improve the biocompatibility of nanoparticles, but whether it reduces the toxicity of GQDs has not yet been evaluated. In this study, the data showed that ferroptotic effects, including cytosolic iron overload (Fig. 9a), GSH depletion (Fig. 9b), MDA enhancement (Fig. 9c), ROS generation (Fig. 9d and e) and oxidative lipid increase (Fig. 9f) were all milder in BV2 cells treated with 100 μg/mL A-GQDs for 24 h than those in cells treated with N-GQDs at the same administration concentration and time. More importantly, the reduced cell viability caused by A-GQDs is slighter than N-GQDs, which could be blocked by pre-treatments of Fer-1 and DFO (Fig. 9g and h).

**Fig. 10:**
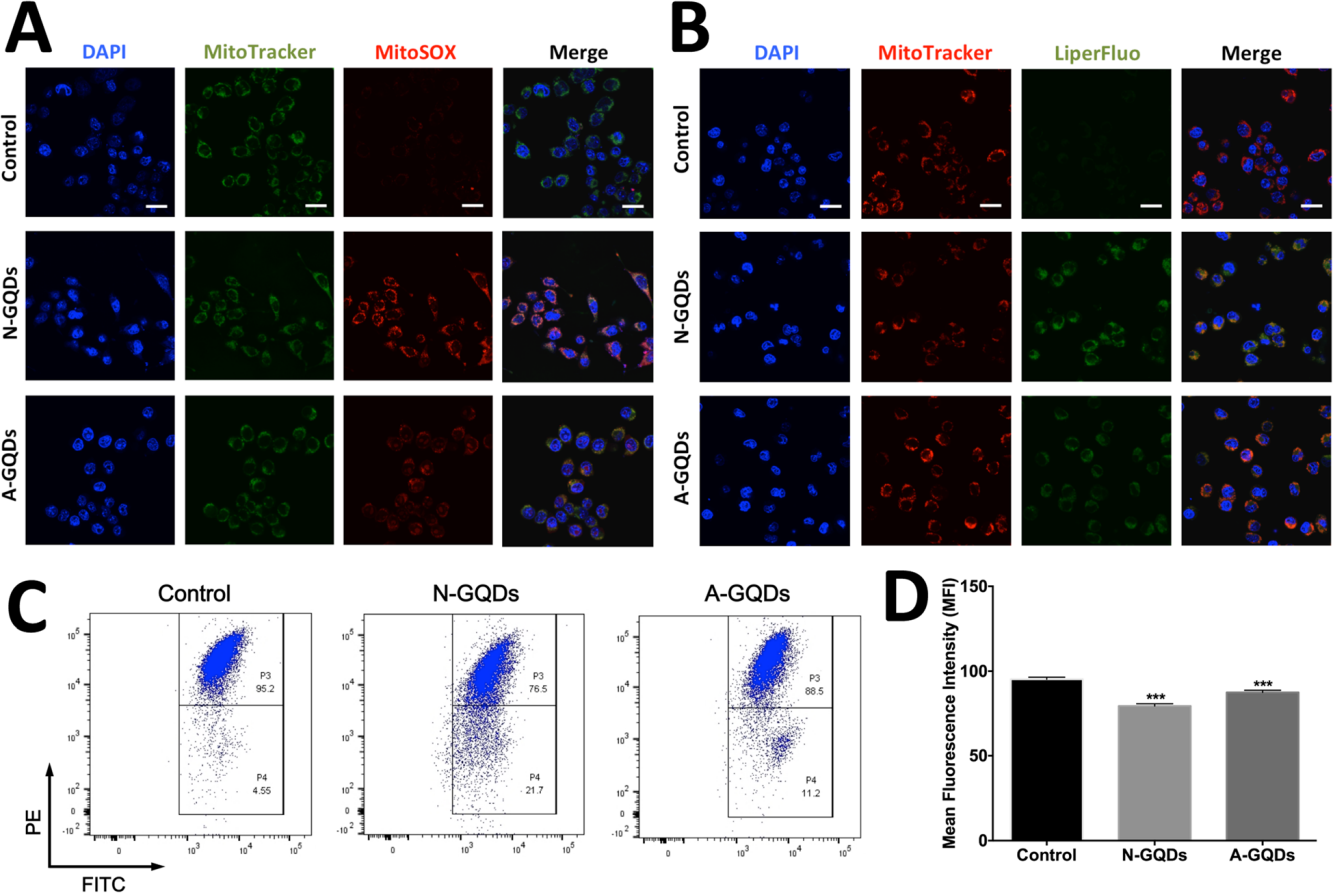
Mitochondrial oxidative stress and dysfunction caused by A-GQDs was milder than N-GQDs. Representative fluorescent images of mitochondrial ROS (mtROS) production (**a**) and mitochondrial lipid peroxidation (**b**) in BV2 cells treated with 100 μg/mL N-GQDs and 100 μg/mL A-GQDs for 24 h. Nuclei are stained by DAPI (blue). mtROS are detected by MitoSOX (red). Oxidative lipid are detected by LiperFluo (green). Mitochondria are marked by MitoTracker (green) and MitoTracker (red), respectively. Merging of the red and green color results in a yellow signal. Scale bars: 20 μm; **c** Representative flow cytometer dot plots of The mitochondrial transmembrane potential (Δψ_mt_) in BV2 cells treated with 100 μg/mL N-GQDs and 100 μg/mL A-GQDs for 24 h were identified by using JC-1; **d** Quantitative results of red fluorescence positive cells from flow cytometer analysis. Data are expressed as the mean ± SE of three independent experiments, performed in triplicate. Statistical significance was determined by one-way ANOVA and Dunnett’s t test (*P < 0.05, **P < 0.01, ***P < 0.001 vs. the control group)

Meanwhile, we found that the productions of ROS (Fig. 10a) and oxidative lipid (Fig. 10b) in mitochondria of BV2 cells treated with A-GQDs were also smaller than those in cells exposed to N-GQDs when the administration concentration and time were same. Even though the MMP in BV2 cells treated with A-GQDs and N-GQDs both significantly changed, the A-GQDs-induced alternation was weaker than N-GQDs (Fig. 10c and d). The data suggested that A-GQDs caused milder mitochondrial oxidative stress and dysfunction than N-GQDs, which might be the mechanisms of A-GQDs exposure resulting in milder ferroptotic effects in BV2 cells.

## Discussion

GQDs are considered as a novel type of QDs with good biocompatibility and provide a bright prospect in the neuroscience application, but the biosafety of them in the CNS has not yet been confirmed. Apart from reported studies showing the blood-brain barrier permeability of GQDs [4], we also observed the fluorescence signal of GQDs in the brain of mice injected with GQDs (Fig. S8). Microglia is an important set of cells to defence foreign chemicals, including carbon-based nanoparticles, in the CNS but to be damaged by them [27]. One study suggested that carbon nanotubes (CNTs) caused cytotoxic effects in microglia-containing cultures instead of neuronal cultures [28].

There have been reported several types of cell death, such as apoptosis, necroptosis and autophagy, are caused by graphene-family nanoparticles, we tried to assess a newly-found PCD called ferroptosis could be induced by GQDs because nanoparticles always cause mixed forms of cell death [13, 29]. Ferroptosis has been identified a form of iron-dependent cell death with lipid peroxidation, and it is mainly occurred in the brain [14, 30]. The microarray data hinted key genes enriched in ferroptosis associated pathways in BV2 cells treated with 100 μg/mL N-GQDs for 24 h.

Firstly, we used two widely reported ferroptosis inhibitors, i.e. a LPO inhibitor Fer-1 and an iron chelating agent DFO, to investigate N-GQDs whether caused ferroptosis in BV2 cells because some researchers have indicated it is necessary to use both lipophilic antioxidants and iron chelators to determine the ferroptosis [23]. The findings showed that pre-treatments of Fer-1 and DFO both effectively prevented decreased cell viability and increased necrotic cells induced by N-GQDs in BV2 cells, which indicated N-GQDs exposure could induce ferroptosis in microglia. It should be noted that N-GQDs exposure simultaneously caused apoptosis in BV2 cells, which was alleviated by Fer-1 but not DFO. The findings indicated the forms of cell death induced by GQDs are mixed and the intracellular oxidative stress could contribute to apoptosis as well, which was confirmed by other antioxidants Trolox and MitoTEMPO.

Meanwhile, the characteristically biological alternations associated with ferroptosis, including iron overload and redox imbalance, were observed in BV2 cells treated with N-GQDs as well. Apart from iron overburden evidenced by a specific fluorescent probe, the disorder of redox homeostasis caused by N-GQDs were indicated by multiple indexes, including GSH depletion, decreased NADPH activity, excessive ROS production and LPO. Similar to other types of graphene-based nanoparticles or QDs, N-GQDs also has been found to be strongly capable of perturbing the redox-sensitive system by inhibiting specific antioxidant enzyme activities in model animals [9, 31].

Additionally, the protection from Fer-1 and DFO against N-GQDs-induced iron accumulation and redox disequilibrium not only authenticated the ferroptosis in BV2 cells caused by N-GQDs but also indicated the association between iron level and redox balance. Pre-treatment of a LPO inhibitor Fer-1 could effectively reverse the iron overload in response to N-GQDs in BV2 cells, because some studies indicated that the LPO is directly attributed to the iron overburden [23, 24]. Furthermore, when BV2 cells pre-treated with an iron chelator DFO, the GSH depletion, increased MDA level and LPO in responses to N-GQDs were also effectively alleviated, which suggested that cytosolic iron burden could contribute to persistent oxidation. Moreover, excessive ROS production caused by N-GQDs inhibited by Fer-1 and DFO could be explained by the facts of iron-mediated ROS generation and LPO via Fenton reaction [32].

After confirming the alternation of gene expression associated with ferroptosis in BV2 cells treated with N-GQDs based on the microarray data, four widely-reported biomarkers of ferroptosis were also used to assess the potential mechanisms of N-GQDs inducing ferroptosis. SLC7A11 is a key component of system X^−^c that is responsible for maintaining redox homeostasis by participating in synthesizing GSH and one of earliest found key regulators in ferroptosis [24, 33]. GPx4 is at the downstream of SLC7A11 and serves a key factor in reducing LPO by converting reduced major antioxidant GSH to GSSG, which is also a confirmed biomarker of ferroptosis [34, 35]. Therefore, the inhibition of expressions of SLC711 and GPx4 could be one reason of GSH depletion in BV2 cells exposed to N-GQDs.

Acyl-CoA synthetase long-chain family member 4 (ACSL4) is a key enzyme of regulating lipid composition, which has been confirmed to contribute to ferroptosis execution [36, 37]. Cyclooxygenase2 (COX2), also known as prostaglandin-endoperoxide synthase (PTGS), has been reported as a monitor of ferroptosis [38]. In this study, the expressions of ACSL4 and COX2 both up-regulated in BV2 cells treated with N-GQDs. In summary, these four proteins were all indexes for redox equilibrium and the alternation of their expression pattern suggested that N-GQDs might instigate ferroptosis in BV2 cells through overwhelming anti-oxidative system that has been reported a key modulator of LPO and resulting in this novel form of cell death [39].

Mitochondria is the major organelle in regulation of iron metabolism and fatty-acid metabolism [40]. Researchers have found some dramatic morphological changes in mitochondria distinction from other forms of PCD, such as mitochondrial shrinkage, in ferroptotic cells [14, 41], which was also observed in mitochondria of BV2 cells treated with N-GQDs. Therefore, the potential involvement of mitochondria in ferroptosis is highly probability, but whether mitochondria play a central role in N-GQDs-induced ferroptosis in microglia remains unclear [26]. In this study, we found that the internalized N-GQDs in BV2 cells was observed in mitochondria, following with obviously mitochondrial impairments, such as broken ridge and collapsed membrane. Meanwhile, the enhancements in iron level, ROS production and oxidative lipid in mitochondria of BV2 cells exposed to N-GQDs were all highlighted by mitochondria targeted indicators. Furthermore, the dissipation of the MMP indicating mitochondrial dysfunction could be associated with the direct damage from N-GQDs or the excessive ROS production in mitochondria [25].

There have been studies suggesting that mitochondrial iron level increased in cells treated with several ferroptosis inducers, such as eratin, doxorubicin and RSL3 [25, 42, 43]. Taken Fenton reaction into consideration, increased mitochondrial iron inevitably leaded to the mitochondrial ROS accumulation, which has been reported to play momentous role in regulating various forms of PCD [44, 45]. ROS could be able to induce LPO by reacting with the polyunsaturated fatty acids of lipid membranes, because mitochondrial fatty acid metabolism provides the specific lipid precursor required for lipid oxidation [26]. Taken all together, the findings suggested that the increased mitochondrial ROS generation caused by N-GQDs might act as an important contributing factor to ferroptosis in BV2 cells.

The intracellular excessive ROS generation, an indicator of oxidative stress, has been reported being essential for the occurrence of ferroptosis [46]. In order to assess the causal relationship of mitochondrial ROS and ferroptosis, BV2 cells were treated with a total antioxidant Trolox and a mitochondrial targeted ROS scavenger MitoTEMPO before they were exposed to N-GQDs. When both Trolox and MitoTEMPO blocked cytosolic and mitochondrial ROS production caused by N-GQDs, they also rescued cell viability in BV2 cells. Moreover, the iron overload and LPO in mitochondria as well as GSH depletion, LPO and alternation of expression pattern of ferroptosis biomarkers induced by N-GQDs were all impeded by Trolox and MitoTEMPO, and the protective capacity of Trolox and MitoTEMPO presented no obvious difference.

Herein, the increased mitochondrial antioxidant activity benefiting from MitoTEMPO effectively blocked ferroptosis induced by N-GQDs in microglia, which indicated that mitochondrial oxidative stress could be a critical step in the N-GQDs-caused execution of ferroptotic cell death. Similar to two ferroptosis inhibitors, i.e. Fer-1 and DFO, a total and a mitochondrial targeted ROS scavenger confer the protective effects against N-GQDs-induced iron overload and redox imbalance not only in the whole BV2 cells but also in the mitochondria. Since only increasing the mitochondrial antioxidant capacity was capable of effectively alleviating the ferroptosis caused by N-GQDs in BV2 cells, the underlying mode of action to explain the N-GQDs-induced ferroptosis could be through the mitochondrial oxidative stress.

As we known, Chemical modification with amino group is a way to improve the biocompatibility of grapheme-based nanoparticles [47], but it is still not certain this better biocompatibility results in lower toxicity. In this study, since the PLQYs of A-GQDs, a kind of amino-functionalized GQDs and N-GQDs are similar, it is meaningful to evaluate the ferroptotic effects of them at the same administration concentration and time. Even though A-GQDs exposure at a high dose was capable of inducing ferroptosis in BV2 cells, the A-GQDs-induced enhancements in ferroptotic cell, cytosolic iron level and LPO were weaker than that induced by N-GQDs.

Based on the results of milder generations of ROS and LPO as well as MMP hyperpolarization in mitochondria of BV2 cells treated with A-GQDs than N-GQDs, it seems that the amino functionalized group might alleviate mitochondrial oxidative stress and dysfunction caused by GQDs and result in mild ferroptosis. According to available studies, amino group functionalization is capable of promoting the linkage between graphene materials and organic molecules, which improve graphene-based nanoparticles more suitable for biomedical applications [47–49]. Some researchers also found that amino group could protect cells from oxidative stress [50]. Even though our findings showed that A-GQDs presented lower toxicity associated with ferroptotic damages and mitochondrial oxidative stress in microglia than N-GQDs, the slight toxicity of A-GQDs still could limit their application in the field of biomedicine. Additionally, N-GQDs seem to be superior on the fluorescent stability because it is recently reported that high concentrations of amino groups tended to exhibit shorter lifetimes [6].

The surface chemical modifications could be associated with other physical and chemical properties, such as surface charge and hydrophobic or hydrophilic nanomaterials, to influencing the physiology of cells. For example, the cellular uptake of positively charged nanoparticles results in higher uptake rates and efficiency in various cell types [51]. Otherwise, whether nanoparticles are hydrophobic or hydrophilic that is mainly determined by their surface ligands are critical to the bio-availability and cytotoxicity of nanoparticles [52]. Although there is not enough data to draw a conclusion in the effects of certain physicochemical parameters of GQDs on the physiological interactions of cells, the researching experience and findings could provide valuable references for further study.

However, there are still some unsolved problems about the molecular pathways of N-GQDs inducing mitochondrial oxidative stress and the specific targets of N-GQDs causing ferroptosis, which should be addressed and clarified before allowing them in biomedical applications. Even though the induction of ferroptosis by GQDs could be expected in cancer therapy via inhibiting cancer cell growth like other nanoparticles [53], it is still a probably serious consequence in response to GQDs in normal tissues, especially the brain that is vulnerable to LPO. The findings advance our understanding of cytotoxicity of GQDs in microglia and provide valuable information for risk assessment of this unique nanoparticles.

## Conclusion

In this study, we found that N-GQDs exposure could cause ferroptosis in BV2 cells following with cytosolic iron overload and redox imbalance, which could be impeded by a ferroptosis-specific inhibitor and an iron chelator. Meanwhile, the uptake of N-GQDs by BV2 cells could be accumulated in mitochondria and damage the normal morphology of mitochondria, which was associated with mitochondrial iron overload and redox imbalance. Benefiting from the mitochondria targeted ROS scavenger MitoTEMPO, we found that the damage in mitochondrial antioxidant ability was crucial in the development of ferroptosis in BV2 cells induced by N-GQDs. When considering the influence of chemical surface modification of GQDs in their nano-bio effects, the findings suggested that amino group functionalized GQDs caused less ferroptosis in BV2 cells than N-GQDs, which might be attributed to A-GQDs inducing milder mitochondrial oxidative stress and dysfunction. This study indicates that it is necessary to analyze the biosafety of so called low-toxic QDs in order to ensure an unbiased and mechanism-based risk assessment of nanoparticles.

## Methods

### Reagents

The SLC7A11, GPx4, ACSL4, COX2 and GAPDH primary antibodies and the HRP conjugated anti-rabbit IgG secondary antibody were purchased from ABclonal Technology (Wuhan, China). Acridine orange (AO) dye was purchased from Solarbio, (Beijing, China). C11-BODIPY^581/591^ was purchased from Thermo Fisher Scientific (MA, USA). FerroOrange, Mito-FerroGreen and LiperFluo were purchased from Dojindo Molecular Technology (Japan). MitoTEMPO were purchased from Sigma (MO, USA). 2′,7′-dichlorodihydrouorescein diacetate (DCFH-DA), Mito-Tracker Red, GSH/GSSG assay kit and Lipid Peroxidation MDA Assay Kit were purchased from Beyotime Biotechnology (Shanghai, China). Cell Counting Kit 8, Annexin V-FITC/propidium iodide assay kit, MitoSOX™ Red Mitochondrial Superoxide Indicator and Mito-Tracker Green were purchased from Yeasen Biotechnology (Shanghai, China). Ferrostain-1, Deferoxamine mesylate (DFO) and Trolox were purchased from APExBIO (TX, USA).

### The characterization of N-GQDs and A-GQDs

The N-GQDs and A-GQDs were purchased from XFNANO materials Tech Co., Ltd. (Nanjing, China). The physicochemical properties of both GQDs were evaluated before the study. High-resolution transmission electron microscope (HR-TEM) images were acquired on an electron microscope. The absorption spectra and fluorescence spectra were measured on a UV-2550 spectrometer. The dynamic light scattering (DLS) measurements were carried out on a Malcern Zetasizer Nano ZS instrument.

### Cell lines and treatment

BV2 microglial cell line was obtained from the Shanghai Cell Research Center (Shanghai, China) and maintained in our laboratory using Dulbecco’s modified Eagle’s medium (DMEM) high glucose medium supplemented with 10% fetal bovine serum (FBS) and 100 U/mL Penicillin. The medium was changed in every 1 ~ 2 days. Cultures were maintained at 37 °C in a humidified atmosphere of 95% O_2_ and 5% CO_2_. The exposure concentrations of N-GQDs were chosen as 25, 50 and 100 μg/mL, and A-GQDs was 100 μg/mL, which were based on the comprehensive analysis of results from CCK8 assay and applied concentration [54]. For inhibitor-pretreatment groups, a specific ferroptosis inhibitors Fer-1, an iron chelator DFO, a total antioxidant Trolox and a mitochondria targeted ROS scavenger MitoTEMPO were added to cells for 2 h prior to 100 μg/mL GQDs addition and maintained in the media until the exposure time was over at a final concentration of 500 nM, 5 μM, 100 μM and 10 μM.

### Cell viability assay

Cell viability was determined using the Cell Counting Kit 8 (CCK8) according to the manufacturer’s protocol. The administration concentrations of N-GQDs were 10, 25, 50, 100, 200, 250 and 500 μg/mL and time was 24 h. The CCK8 assay was performed independently in triplicate.

### Cell uptake of GQDs

After exposure to 25, 50 and 100 μg/mL N-GQDs for 24 h, the cellular uptakes of GQDs were measured by using a flow cytometry (FACSCanto II; BD Bioscience) at excitation and emission wavelengths of 488 (438) nm and 525 (405) nm (referred to FICH channel). Meanwhile, the fluorescence intensities of N-GQDs in cells treated with 100 μg/mL N-GQDs for 24 h were measured by a confocal microscope with acridine orange (AO) marking nuclei and Mito-Tracker Green making mitochondria. All the treatments were performed in triplicate in three independent experiments.

### Annexin V-FITC/Propidium iodide staining

The BV2 cells treated with GQDs were distinguished into normal, apoptotic, and necrotic cells by using an Annexin V-FITC/propidium iodide assay kit with a flow cytometry (FACSCanto II; BD Bioscience) based on the manufacturer’s instructions. All the treatments were performed in triplicate in three independent experiments.

### Cellular and mitochondrial iron detection

The levels of iron concentrations in cytoplasm and mitochondria were assessed by using FerroOrange probes and Mito-FerroGreen probes through a fluorescence microscope or a confocal microscope, respectively. When detecting the mitochondrial iron, the Mito-Tracker Red was used to mark mitochondria. All the treatments were performed in triplicate in three independent experiments.

### Determination of GSH/GSSG activity

The intracellular levels of glutathione (GSH) and oxidized glutathione (GSSG) were determined by using commercial GSH/GSSG assay kit following the manufacturer’s instructions. All the treatments were performed in triplicate in three independent experiments.

### Malondialdehyde (MDA) assay

The MDA contents in cells were measured using the commercial Lipid Peroxidation MDA Assay Kit following the manufacturer’s instructions. All the treatments were performed in triplicate in three independent experiments.

### Lipid peroxidation assessed by C11-BODIPY^581/591^ and LiperFluo staining

To perform C11-BODIPY^581/591^, BV2 cells were incubated with PBS containing C11-BODIPY^581/591^ at final concentration of 5 μM for 15 min at 37 °C in the dark and observed by a fluorescence microscope. For LiperFluo staining, cells were incubated with PBS containing Liperfluo at final concentration of 10 μM for 30 min at 37 °C in the dark and observed by a confocal microscope with the Mito-Tracker Red marking mitochondria. All the treatments were performed in triplicate in three independent experiments.

### Measurement of cytosolic reactive oxygen species (ROS) generation

The levels of cytosolic ROS were determined by using DCFH-DA with a flow cytometry (FACSCanto II; BD Bioscience) based on the manufacturer’s instructions. All the treatments were performed in triplicate in three independent experiments.

### Ultrastructure observation of cells

The BV2 cells were fixed in PBS containing 2.5% glutaradehyde, followed by the standard fixation protocol described in a previous study [55]. Finally, the ultrathin sections of cells were observed by a transmission electron microscope.

### Measurement of mitochondrial ROS (mtROS) generation

The levels of mtROS were determined by using MitoSOX™ red mitochondrial superoxide indicator with Mito-Tracker Green marking mitochondria and a confocal microscope according to the manufacturer’s instructions. All the treatments were performed in triplicate in three independent experiments.

### Mitochondrial injury assessment

The change on mitochondrial membrane potential (MMP) was detected by using 5,5′,6,6′-tetrachloro-1,1′,3,3′-tetraethyl-benzimidazolcarbocyanine (JC-1) with a flow cytometry (FACSCanto II; BD Bioscience) based on the manufacturer’s instructions. All the treatments were performed in triplicate in three independent experiments.

### Microarray assay

Two groups (three parallels in each group), i.e. control and 100 μg/mL N-GQDs, were designed for detection of the differentially expressed genes. Total RNAs extracted by Trizol reagent was quantified and assessed by a NanoDrop ND-2000 spectrophotometer (Thermo Scientific) at A260/A280 nm, and an Agilent Bioanalyzer 2100 (Agilent Technologies), respectively. The microarray analysis was completed by OE Biotech. Co., Ltd. (Shanghai, China), and the sample labelling, microarray hybridization and washing were strictly performed in accordance with standard protocols. The detailed process is described in a previous study [56].

### Quantitative real-time reverse-transcription polymerase chain reaction (qRT-PCR) analysis

Equal quantities of total RNA of BV2 cells were used to do qRT-PCR analysis, which was carried out as above description. The qRT-PCR primers were designed by software Primer Premier based on National Center for Biotechnology Information (NCBI) (Table S1). The relative quantities of mRNA were normalized against the mRNA of reference gene gapdh. Three replicates were conducted for each qRT-PCR analysis.

### Western blotting

Total protein of BV2 cells was extracted to do western blotting analysis, which was carried out following the protocol described in a previous study [57]. The same samples were ran three times and the experiment was repeated independently at least three times.

### Data analysis

All data were displayed as the mean ± standard deviation (SD). Statistical analysis was performed using Graphpad Prism 6.0 Software. One-way analysis of variance (ANOVA) was used to determine the statistical significance between control and exposed groups, followed by the Dunnett’s t test to determine the significance of differences between groups. Probability levels of < 0.05, < 0.01 and < 0.001 were considered statistically significant.

## Supplementary information

**Additional file 1.**

## Data Availability

The datasets during and/or analyzed during the current study available from the corresponding author on reasonable request.
