# Supplementary material for: Induction of ferroptosis in response to graphene quantum dots through mitochondrial oxidative stress in microglia
Source: Part Fibre Toxicol. 2020 Jul 11;17:30. doi: 10.1186/s12989-020-00363-1 (PMC7353734; doi:10.1186/s12989-020-00363-1)
Supplement: Supplementary file 1 — Additional file 1. [file 12989_2020_363_MOESM1_ESM.docx]

**Supporting information**

**Table S1:** Designed qRT-PCR primers of genes.

| **Gene name** | **Designed qRT-PCR primers** | |
| --- | --- | --- |
|  | Forward | Backward |
| ptgs2 | TGCGATGCTCTTCCGAGCTG | TTTGGGGTGGGCTTCAGCAG |
| gpx4 | AAGAACGGCTGCGTGGTGAA | CACAAGGCAGCCAGGGTGAA |
| slc7a11 | CGGTGGTGTGTTCGCTGTCT | GTGCCGGGATGAAGAGAGGC |
| nox1 | GACAGGTGCCTTTGCCTGGT | AAACCCCCACCGCAGACTTG |
| ptges | TTGCCCACAAGGTCTGCCTG | ACTGTCCCTTGCTTGGTGCC |
| nqo1 | TTGGGGTGCCAGCCATTCTG | GGTGGAGTGTGGCCAATGCT |
| acsl4 | TGGACCCCCGAGACTGGTTT | ACCATCAGGCCACCTCCTGT |
| fth1 | GACGTTCTCGCCCAGAGTCG | CCGCGTCCTGGTGGTAGTTC |
| tfrc | GAAACCAGTTGGCCCTGGCT | GTGTAGGTCCAACCCCGCAC |
| ftl | CTCCTCGCTGCCTTCAGCTC | GTTCACGGCAGCTTCCACCT |
| gapdh | CCTGTTCCAGAGACAGCCGC | GCGCCCAATACGGCCAAATC |

**Table S2:** The summary of physicochemical characteristics of N-GQDs and A-GQDs

| QDs | Mean size by TEM (nm) | Mean size by DLS in DI water (nm) | Mean size by DLS in DMEM (nm) | ξ-potential in DI water (mV) | ξ-potential in DMEM (mV) | Excitation peak in DI water (nm) | Excitation peak in DMEM (nm) | Emission peak in DI water (nm) | Emission peak in DMEM (nm) | PLQY (%) |
| --- | --- | --- | --- | --- | --- | --- | --- | --- | --- | --- |
| N-GQDs | ~3 | 3.9 | 3.1 | -9.9 | -12.2 | 350 | 380 | 420 | 480 | ~20 |
| A-GQDs | ~4 | 3.8 | 3.4 | -25.9 | -14.3 | 380 | 380 | 420 | 420 | ~20 |


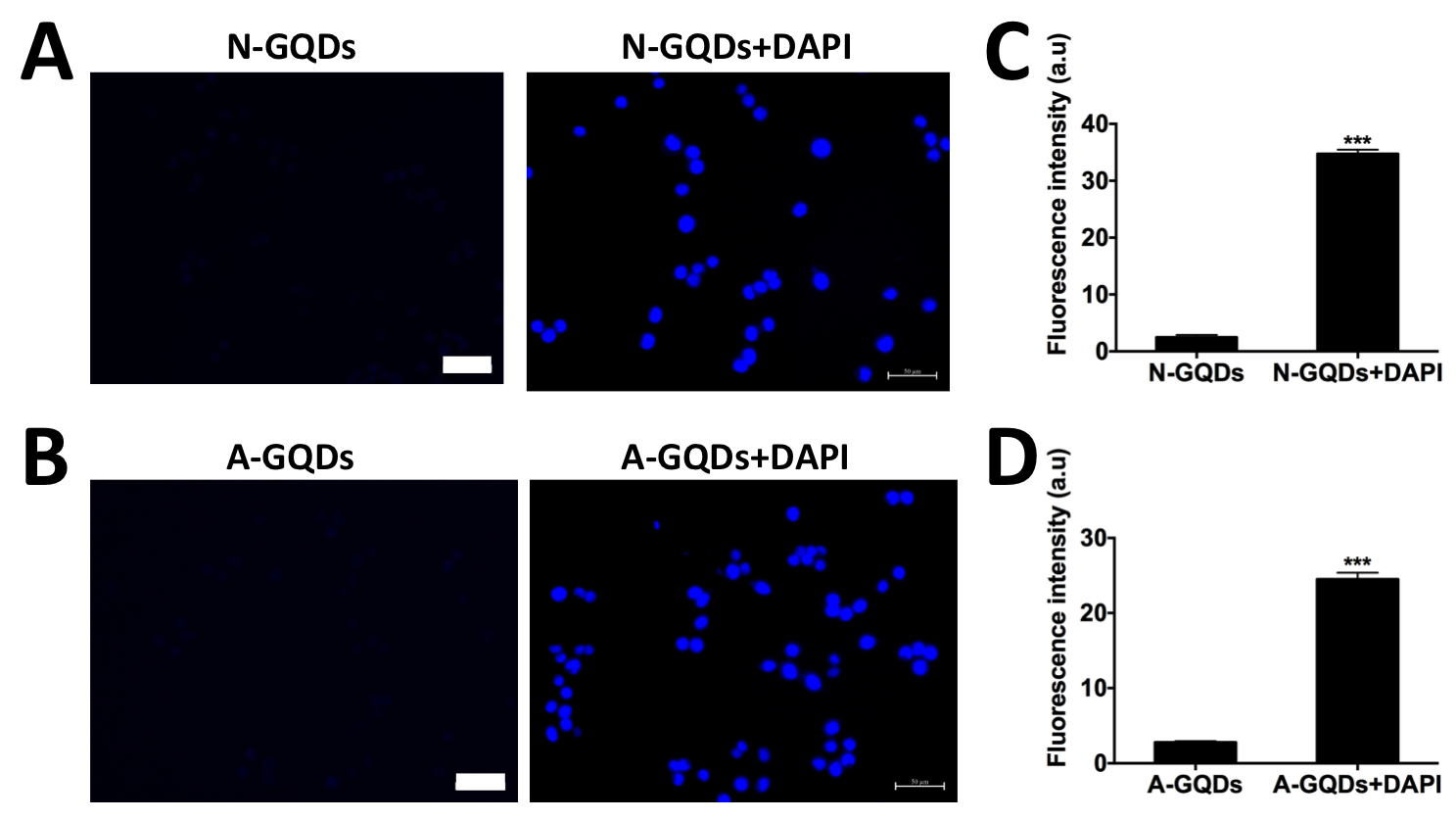


**Figure S1:** The high content screening (HCS) images of BV2 cells treated with 100 µg/mL N-GQDs (A) and 100 µg/mL A-GQDs (B) in the absence and presence of DAPI at 347 nm excitation and 483 nm emission. Scale bars: 50 µm; (C, D) Quantitative results of fluorescence intensities in images A and B. It is shown that fluorescence intensity in cells treated with N-GQDs or A-GQDs were only 7.14% and 11.37% of that in N-GQDs- or A-GQDs-treated cell after staining by DAPI, respectively. Thereby, the fluorescence intensity of N-GQDs and A-GQDs at 347 nm excitation and 483 nm emission can be ignored.


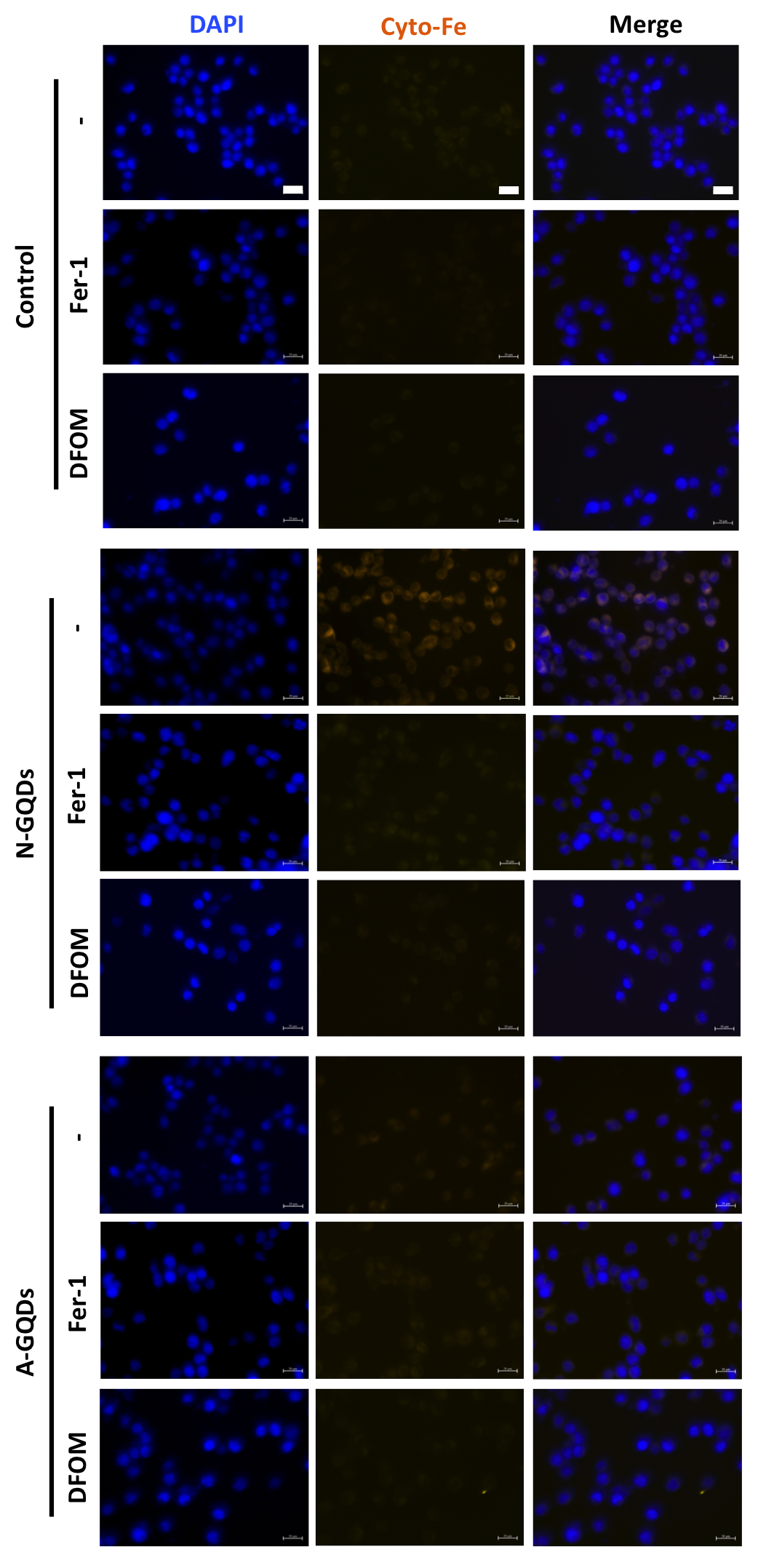


**Figure S2:** Representative fluorescent images of intracellular iron level in BV2 cells exposed to 100 µg/mL N-GQDs and 100 µg/mL A-GQDs for 24 h pretreated with/without Ferrostain-1 and DFMO were identified by using FerroOrange (orange). Nuclei are stained by DAPI (blue). Scale bars: 50 µm.


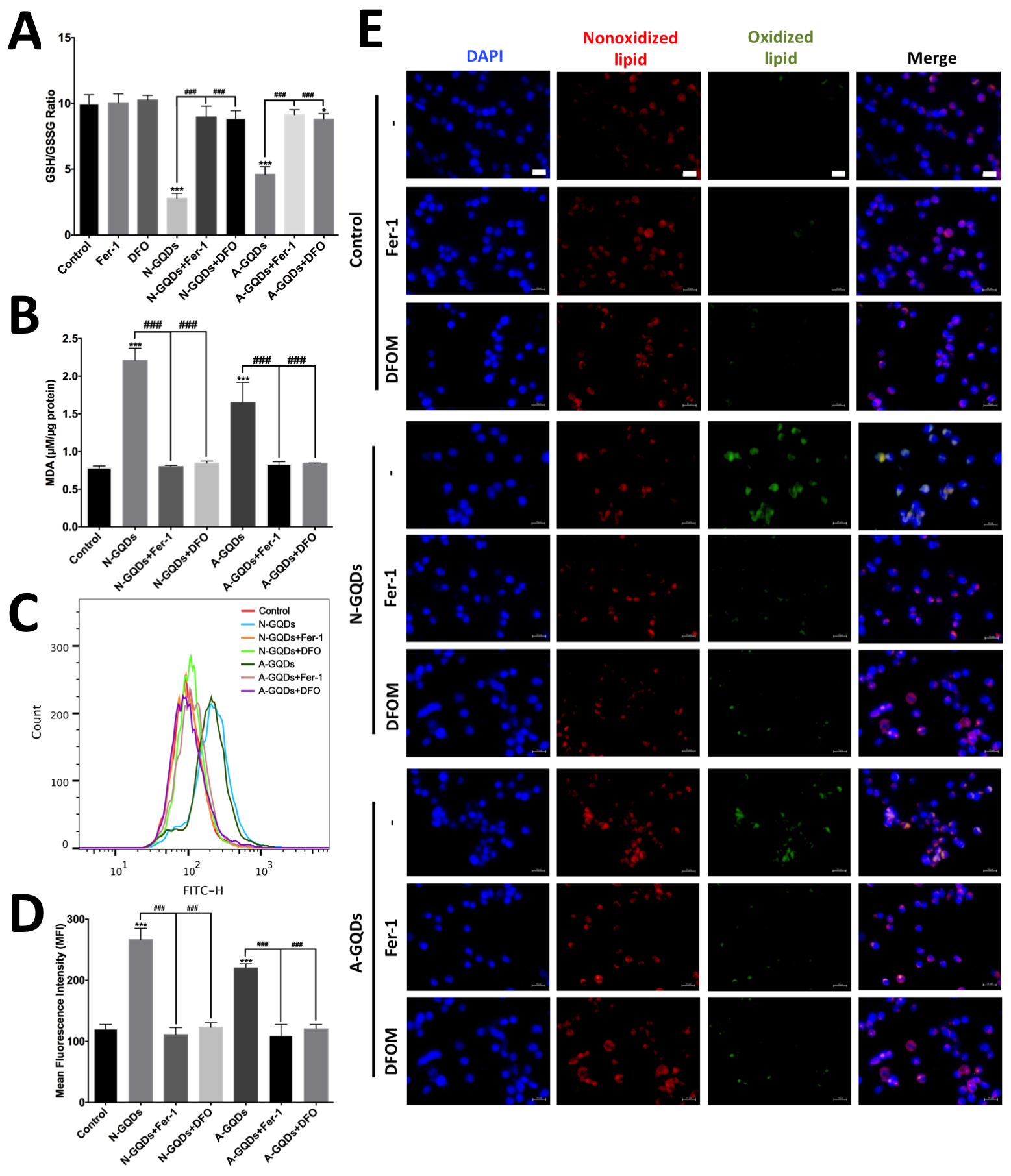


**Figure S3:** Ferroptosis inhibitors alleviated redox imbalance caused by GQDs in BV2 cells. The GSH/GSSG ratio (A) and the MDA content (B) in BV2 cells exposed to 100 µg/mL N-GQDs and 100 µg/mL A-GQDs for 24 h pretreated with/without Fer-1 and DFO were measured by ELISA; (C) Representative FITC fluorescence histogram plot of cytosolic ROS production in BV2 cells treated with 100 µg/mL N-GQDs and 100 µg/mL A-GQDs for 24 h pretreated with/without Ferrostain-1 and DFMO were identified by DCFH-DA; (D) Quantitative results of mean fluorescence intensity (MFI) from flow cytometer analysis; (E) Representative fluorescent images of lipid ROS in BV2 cells exposed to 100 µg/mL N-GQDs and 100 µg/mL A-GQDs for 24 h pretreated with/without Fer-1 and DFO were identified by using C11BODIPY^581/591^. Nonoxidized lipid is represented in red, oxidized lipid is in green, nuclei are stained by DAPI (blue), merging of the red and green color results in a yellow signal. Scale bars: 50 µm. Data are expressed as the mean ± SE of three independent experiments, performed in triplicate. Statistical significance was determined by one-way ANOVA and Dunnett’s t test (**P* < 0.05, ***P* < 0.01, ****P* < 0.001 vs. the control group; #*P* < 0.05, ##*P* < 0.01, ###*P* < 0.001 vs. the 100 µg/mL N-GQDs group).


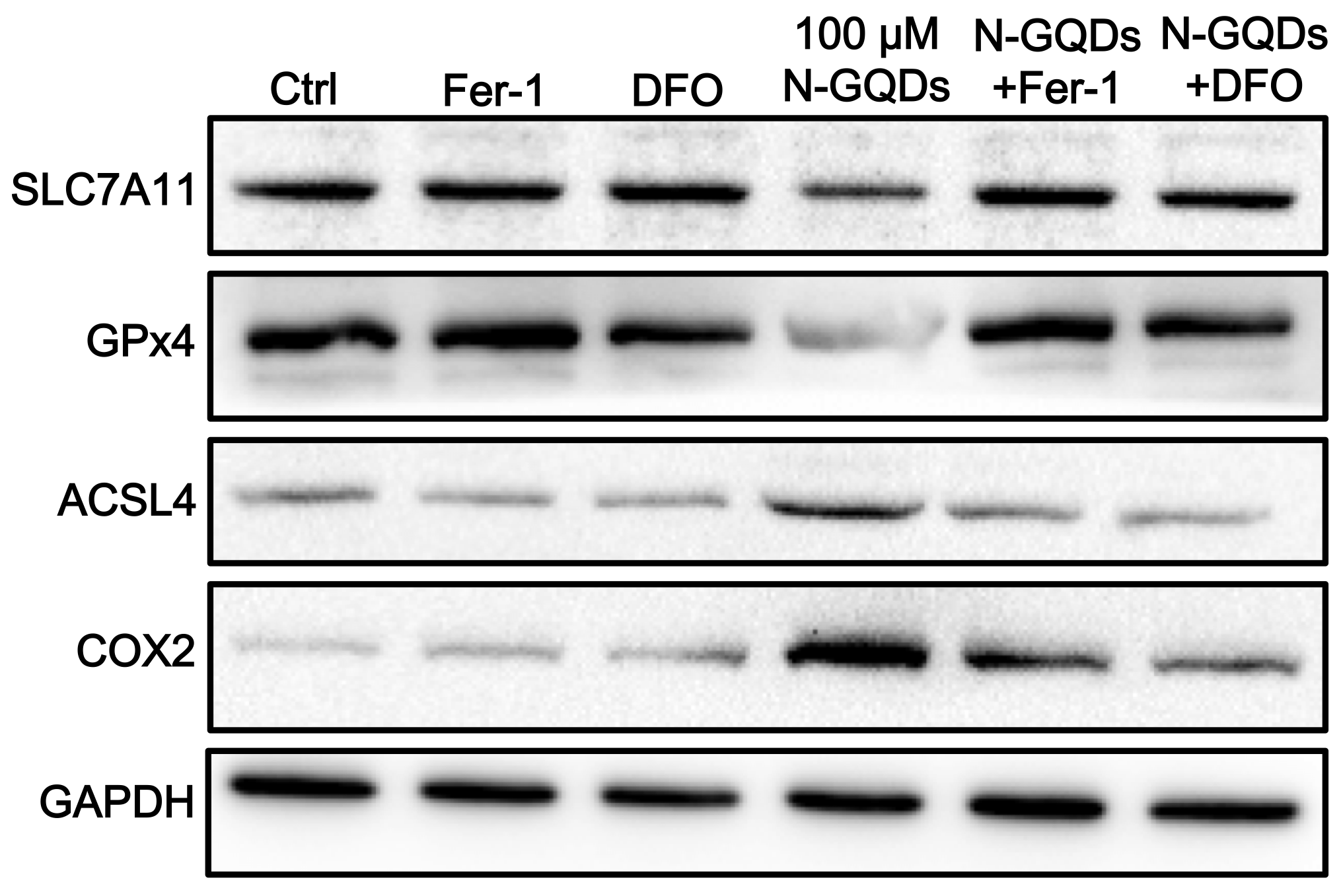


**Figure S4:** The expressions of ferroptosis marker proteins SLC7A11, GPX4, ACSL4 and COX2 in BV2 cells exposed to 100 µg/mL N-GQDs for 24 h pretreated with/without Fer-1 and DFO were determined by western blotting analysis.


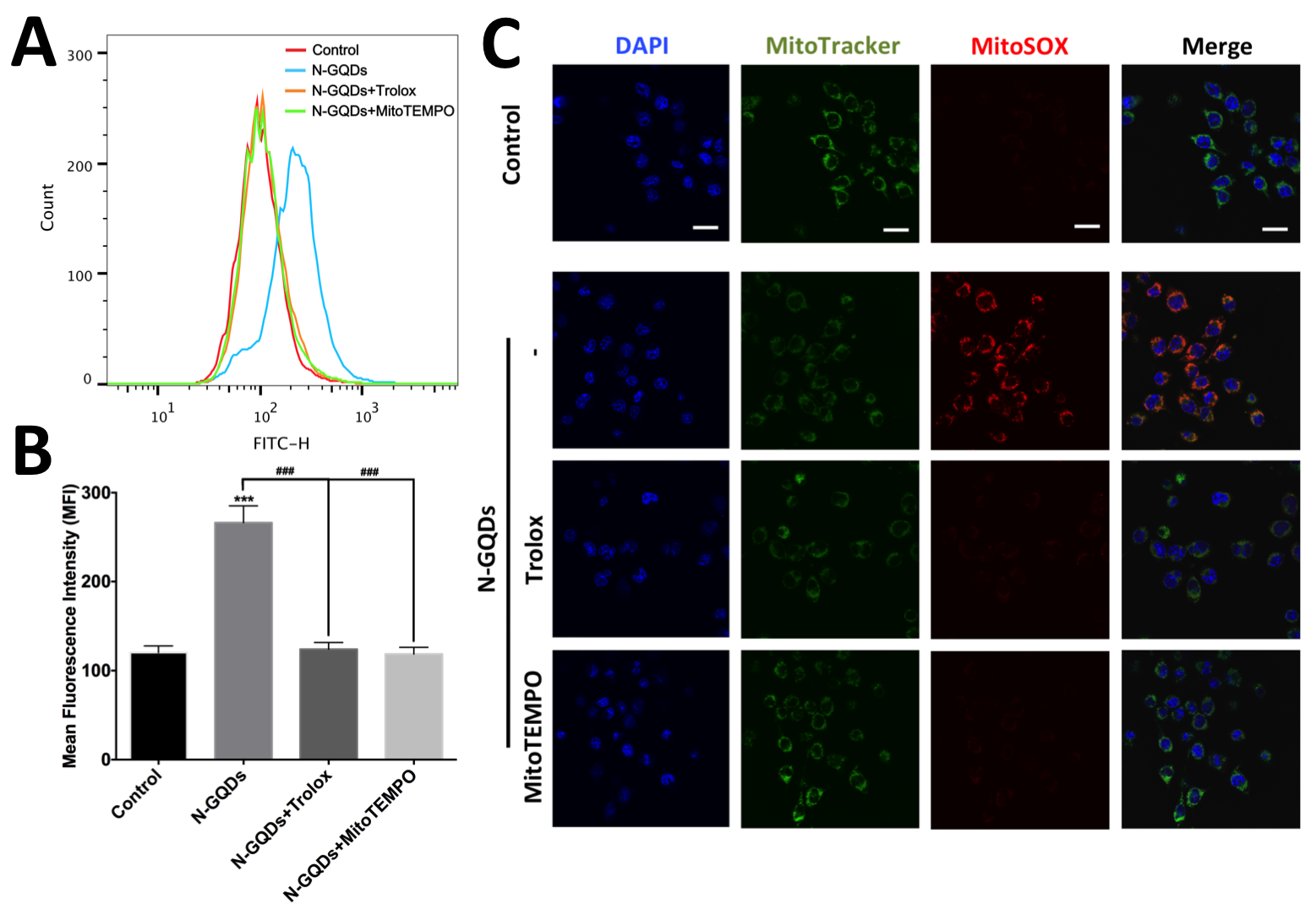


**Figure S5:** The ROS scavengers alleviated the ROS generation in cytoplasm and mitochondria of BV2 cells caused by N-GQDs. (A) Representative FITC fluorescence histogram plot of cytosolic ROS production in BV2 cells treated with 100 µg/mL N-GQDs for 24 h pretreated with/without Trolox and MitoTEMPO were identified by DCFH-DA; (B) Quantitative results of mean fluorescence intensity (MFI) from flow cytometer analysis; (C) Representative fluorescent images of mitochondrial ROS (mtROS) production in BV2 cells treated with 100 µg/mL N-GQDs for 24 h pretreated with/without Trolox and MitoTEMPO were identified by MitoSOX (red) and MitoTracker (green). Nuclei are stained by DAPI (blue), merging of the red and green color results in a yellow signal. Scale bars: 20 µm. Statistical significance was determined by one-way ANOVA and Dunnett’s t test (**P* < 0.05, ***P* < 0.01, ****P* < 0.001 vs. the control group; #*P* < 0.05, ##*P* < 0.01, ###*P* < 0.001 vs. the 100 µg/mL N-GQDs group).


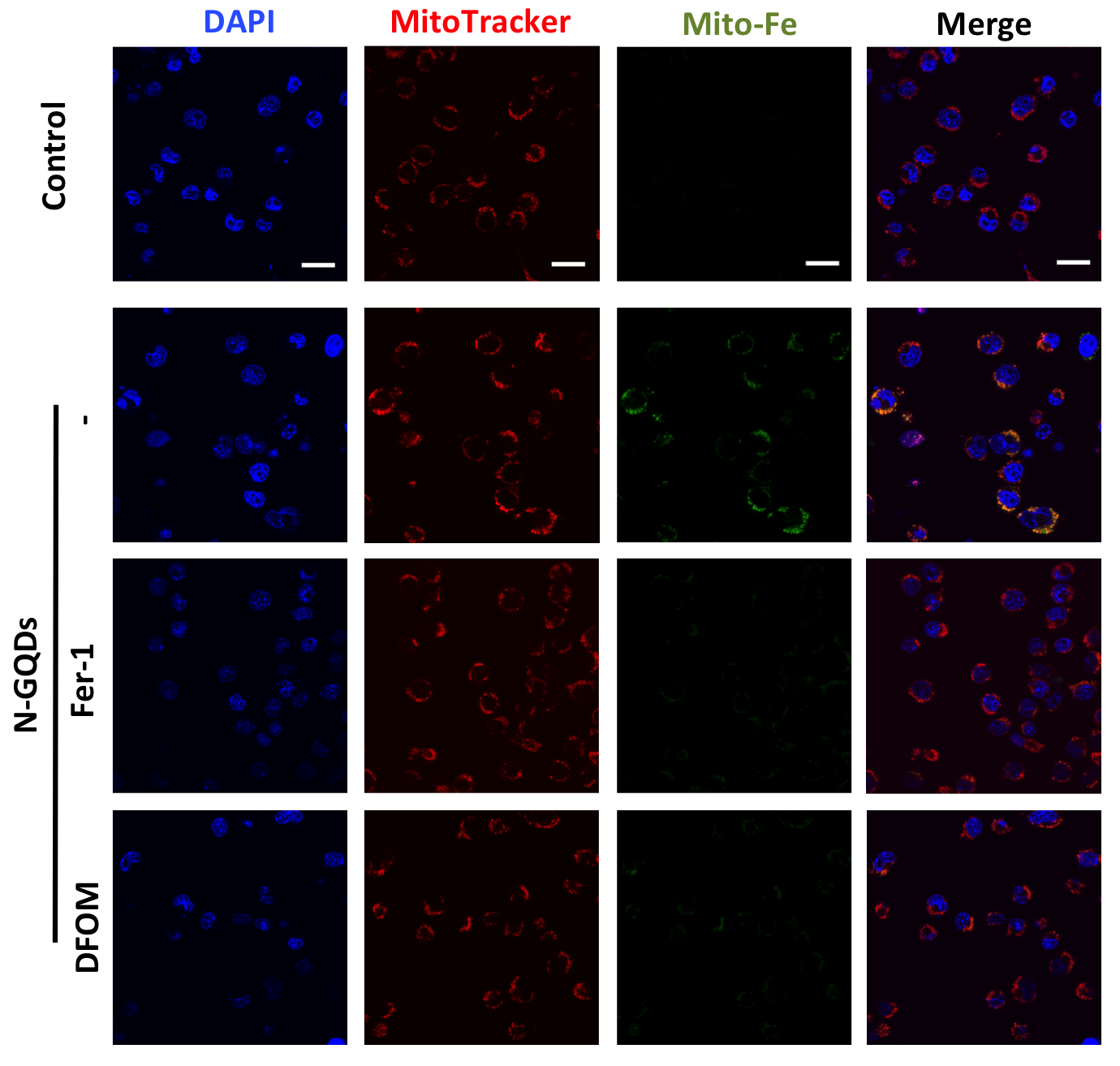


**Figure S6:** Representative fluorescent images of mitochondrial iron level in BV2 cells exposed to 100 µg/mL N-GQDs for 24 h pretreated with/without Ferrostain-1 and DFMO were identified by using Mito-FerroGreen (green) and MitoTracker (red). Nuclei are stained by DAPI (blue). Scale bars: 20 µm.


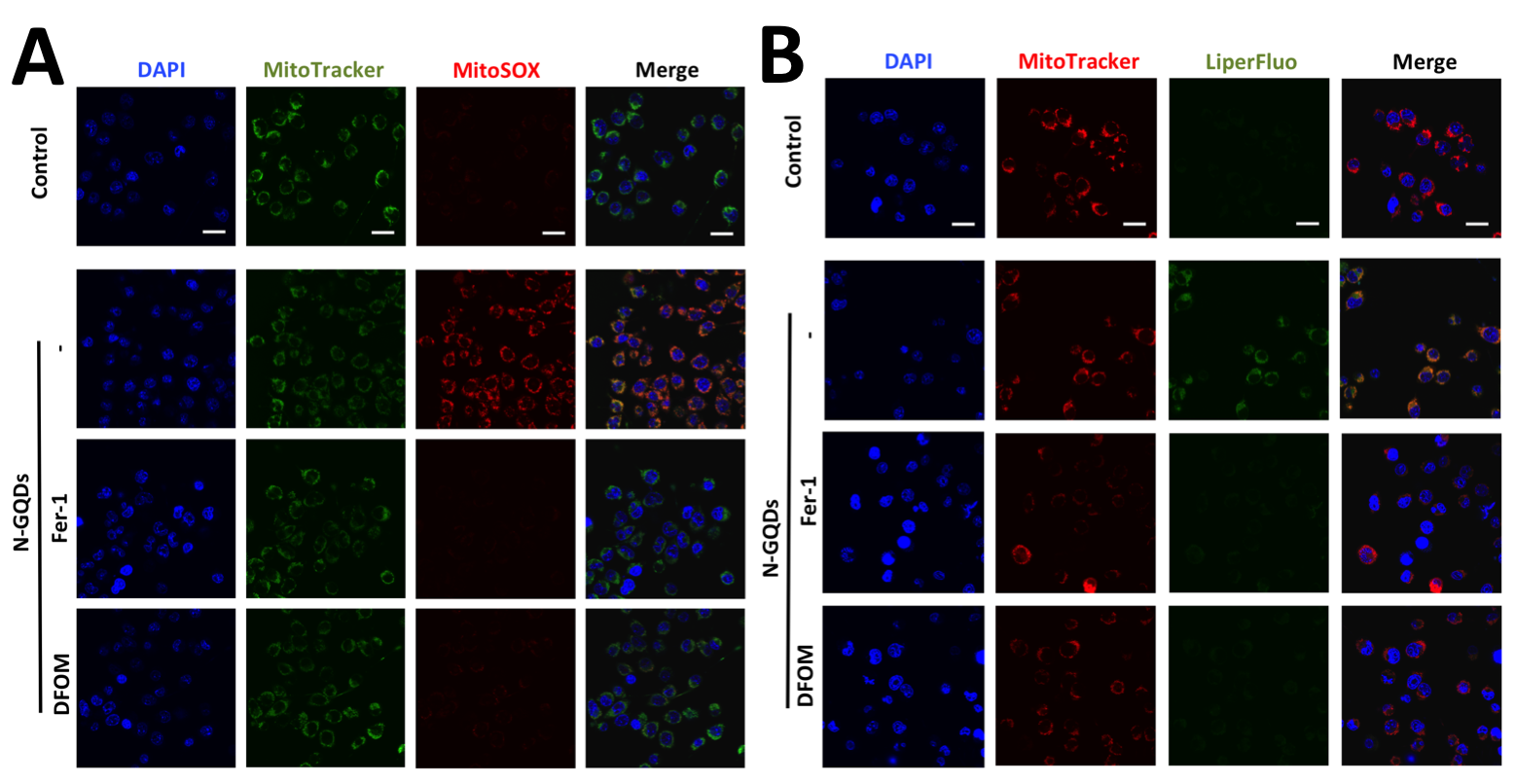


**Figure S7:** Ferroptosis inhibitors alleviated mitochondrial oxidative stress caused by N-GQDs in BV2 cells. Representative fluorescent images of mitochondrial ROS (mtROS) production (A) and lipid peroxidation (B) in BV2 cells exposed to 100 µg/mL N-GQDs for 24 h pretreated with/without Fer-1 and DFO. Nuclei are stained by DAPI (blue). mtROS were detected by MitoSOX (red). Oxidative lipid were detected by LiperFluo (green). Mitochondria were marked by MitoTracker (green) and MitoTracker (red), respectively. Merging of the red and green color results in a yellow signal. Scale bars: 20 µm.


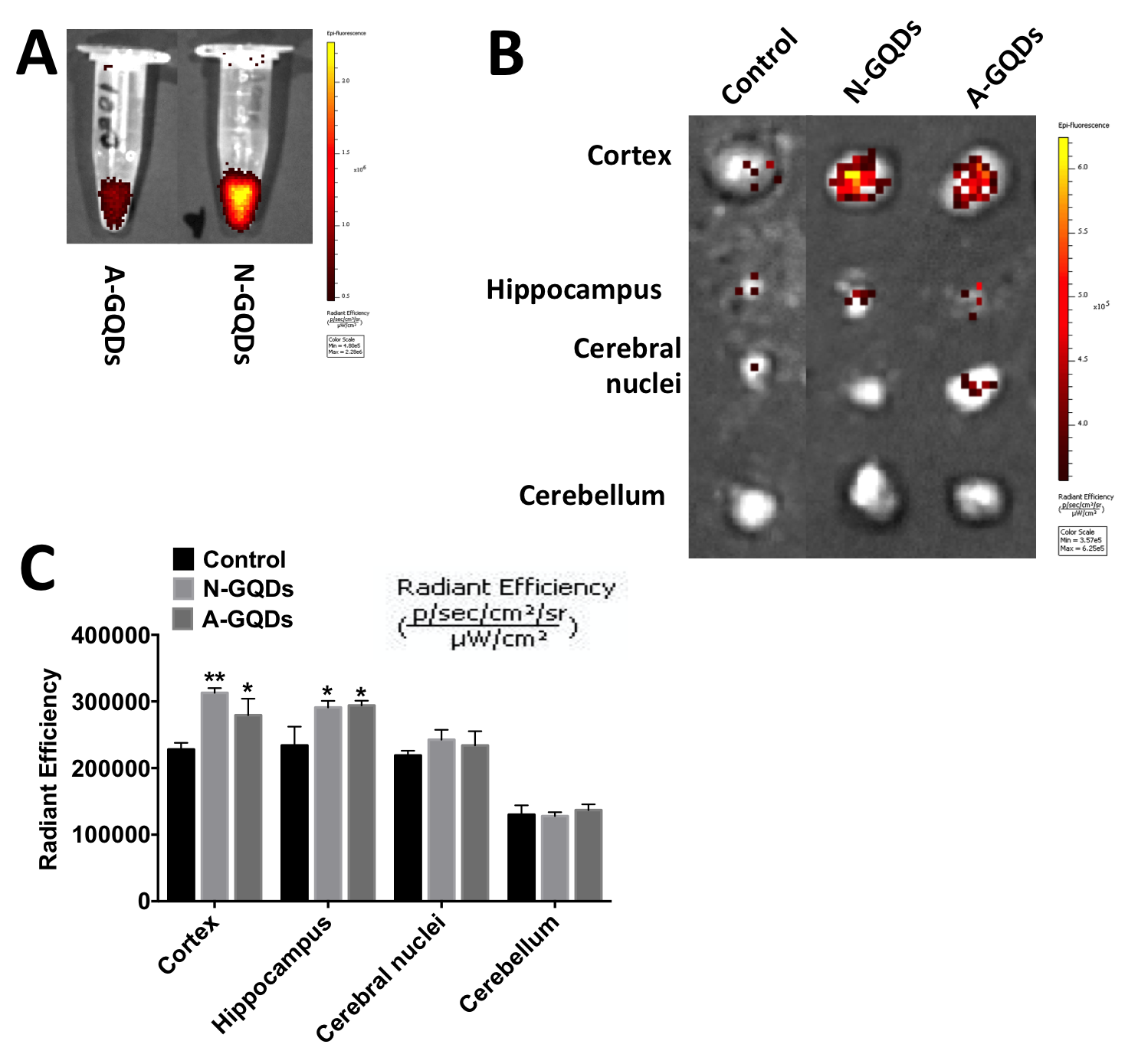


**Figure S8:** The distribution of the N-GQDs and A-GQDs in different brain regions of mouse intravenously injected with 10 mg/kg Body Weight GQDs through the tail for 3 h. (A) Representative fluorescent images of N-GQDs and A-GQDs; (B) Representative fluorescent images of four brain regions, *i.e.* cortex, hippocampus, cerebral nuclei, cerebellum, of mice treated with N-GQDs and A-GQDs; (C) The radiant efficiency of the different brain regions (n=3). Statistical significance was determined by one-way ANOVA and Dunnett’s t test (**P* < 0.05, ***P* < 0.01, ****P* < 0.001 vs. the control group).
